# Supplementary material for: A dynamic feedback loop between retrograde sterol transport and TORC2 controls adaptation of the plasma membrane to stress
Source: EMBO J. 2025 Nov 13;44(24):7541–64. doi: 10.1038/s44318-025-00618-7 (PMC12705765; doi:10.1038/s44318-025-00618-7)
Supplement: Supplementary file 1 — Appendix [file 44318_2025_618_MOESM1_ESM.pdf]

Appendix for

A dynamic feedback loop between retrograde sterol transport and TORC2 controls adaptation of the plasma membrane to stress

Maria G. Tettamanti[1,2], Paulina Nowak[1,2], Beata Kusmider[1], Jennifer M. Kefauver[1,4¤], Vincent Mercier[3], Aurélien Roux[2*], Robbie Loewith[1*]

Table of contents

Appendix Table S1 2

Appendix Table S1: Drug / substance stocks used in this study

substance	stock
Rapamycin	200 µM in DMSO
CK-666	125 mM in DMSO
Atorvastatin	20 mM in DMSO
Fluconazole	20 mM in DMSO
Carnitine	10 mM in PBS
Palmitate (C16)	10 mM in DMSO
Palmitoylglycine (C16-Glycine)	10 mM in DMSO
Palmitoylglycerol (C16-Glycerol)	10 mM in DMSO
Palmitoylcholine (C16-Choline)	10 mM in DMSO
PAF	10 mM in DMSO
LauroylCarnitine (C12-C)	10 mM in MeOH
MyristoylCarnitine (C14-C)	10 mM in MeOH
PalmitoylCarnitine (C16-C)	10 mM in DMSO, or MeOH (only SAR screen)
StearoylCarnitine (C18-C)	10 mM in MeOH
OleoylCarnitine (C18:1-C)	10 mM in MeOH
L-*erythro*-DHS	5 mM in DMSO
D-*erythro*-DHS	5 mM in DMSO
PHS	10 mM in DMSO
