# Supplementary figures and images for: A dynamic feedback loop between retrograde sterol transport and TORC2 controls adaptation of the plasma membrane to stress
Source: EMBO J. 2025 Nov 13;44(24):7541–64. doi: 10.1038/s44318-025-00618-7 (PMC12705765; doi:10.1038/s44318-025-00618-7)

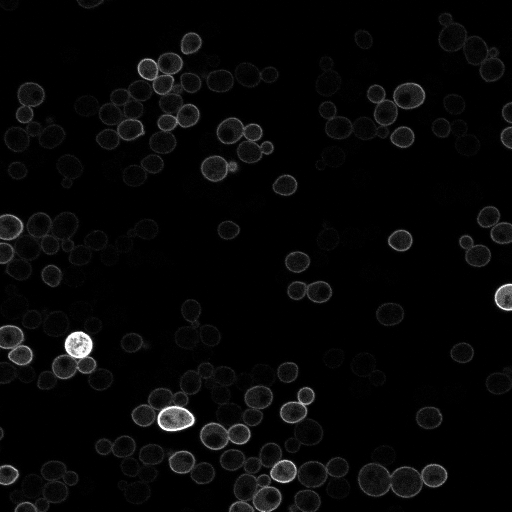

Supplement: Supplementary file 15 — Source data Fig. 1 [file 44318_2025_618_MOESM15_ESM.zip › Figure 1/1C/DHS_t30.tif]

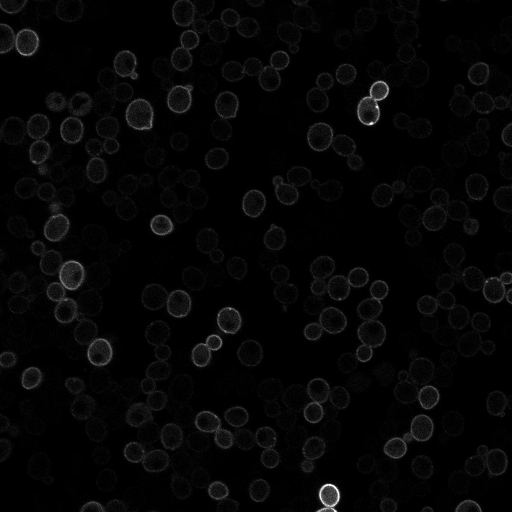

Supplement: Supplementary file 15 — Source data Fig. 1 [file 44318_2025_618_MOESM15_ESM.zip › Figure 1/1C/DHS_t5.tif]

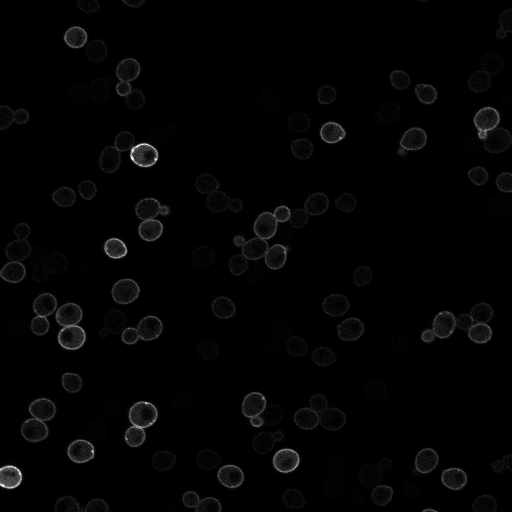

Supplement: Supplementary file 15 — Source data Fig. 1 [file 44318_2025_618_MOESM15_ESM.zip › Figure 1/1C/PHS_t30.tif]

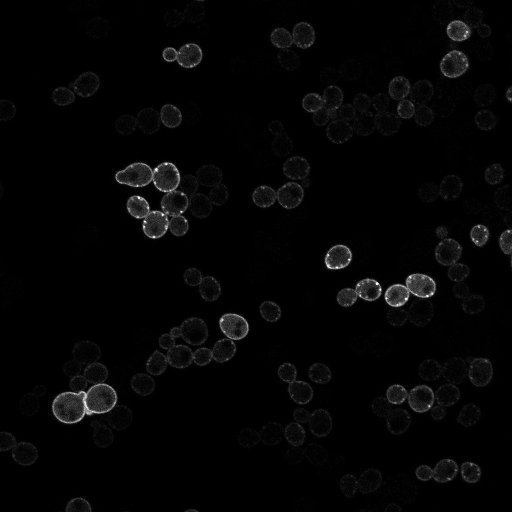

Supplement: Supplementary file 15 — Source data Fig. 1 [file 44318_2025_618_MOESM15_ESM.zip › Figure 1/1C/PHS_t5.tif]

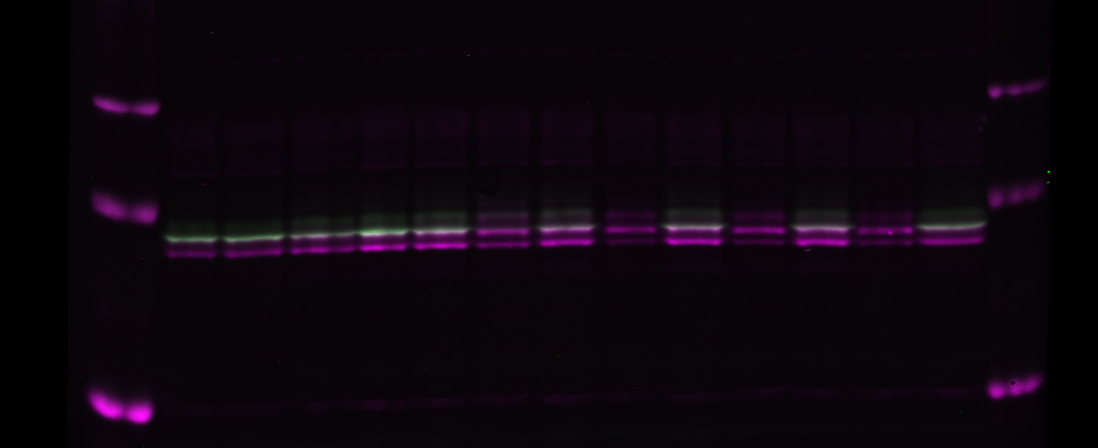

Supplement: Supplementary file 15 — Source data Fig. 1 [file 44318_2025_618_MOESM15_ESM.zip › Figure 1/1D/uncropped WBs/20210714.tif]

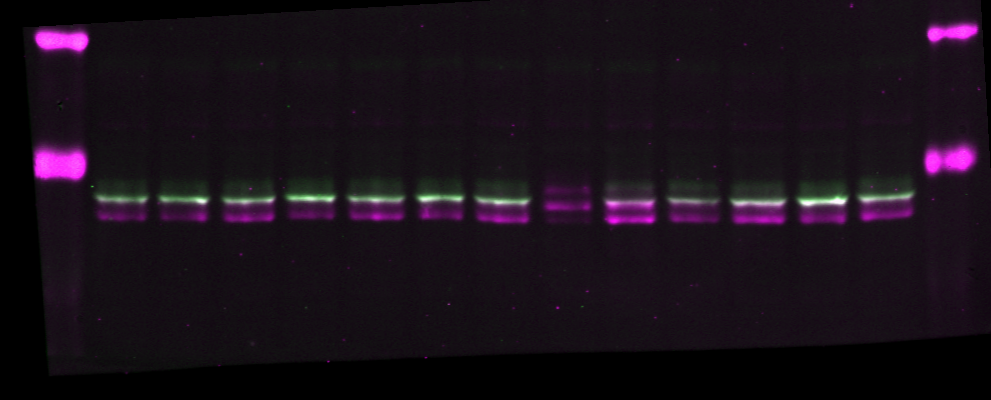

Supplement: Supplementary file 15 — Source data Fig. 1 [file 44318_2025_618_MOESM15_ESM.zip › Figure 1/1D/uncropped WBs/20210804B.tif]

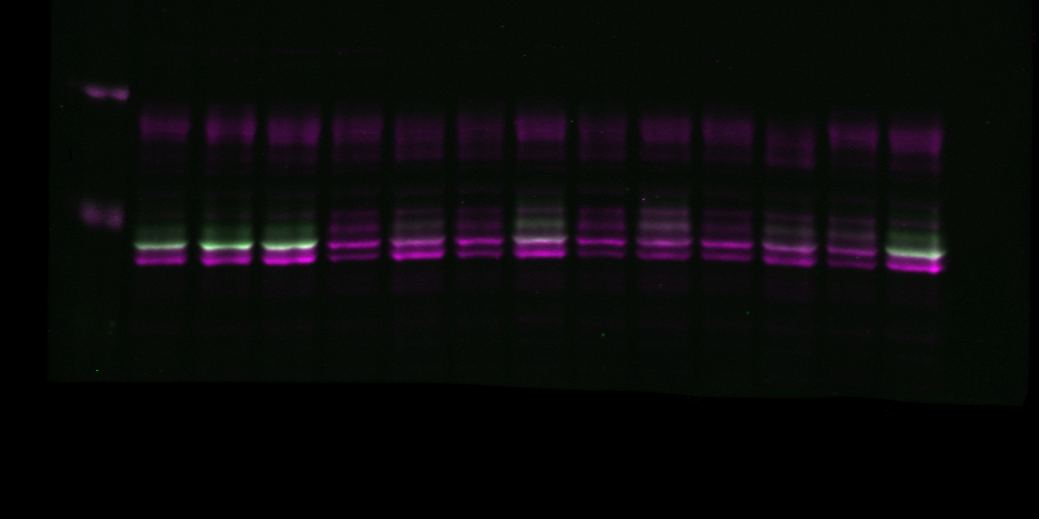

Supplement: Supplementary file 15 — Source data Fig. 1 [file 44318_2025_618_MOESM15_ESM.zip › Figure 1/1D/uncropped WBs/20210825B.tif]

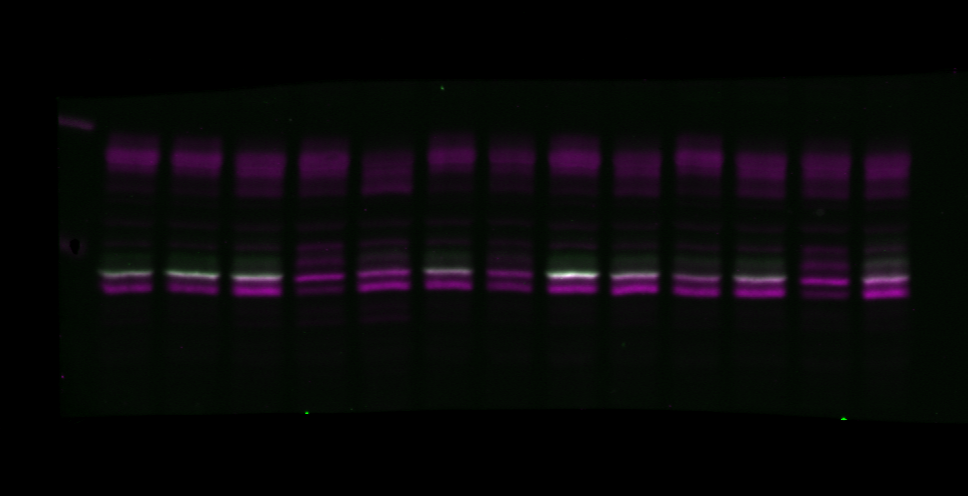

Supplement: Supplementary file 15 — Source data Fig. 1 [file 44318_2025_618_MOESM15_ESM.zip › Figure 1/1D/uncropped WBs/20210915.tif]

# Experiment 20210804B

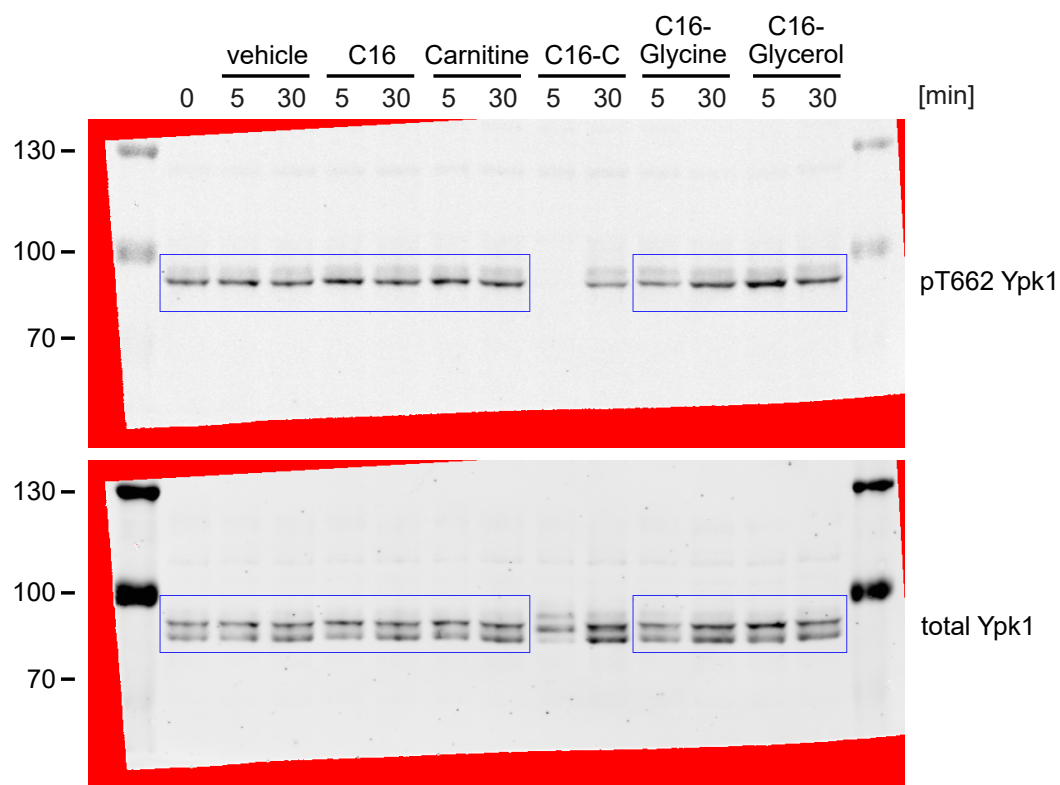

# Experiment 20210714

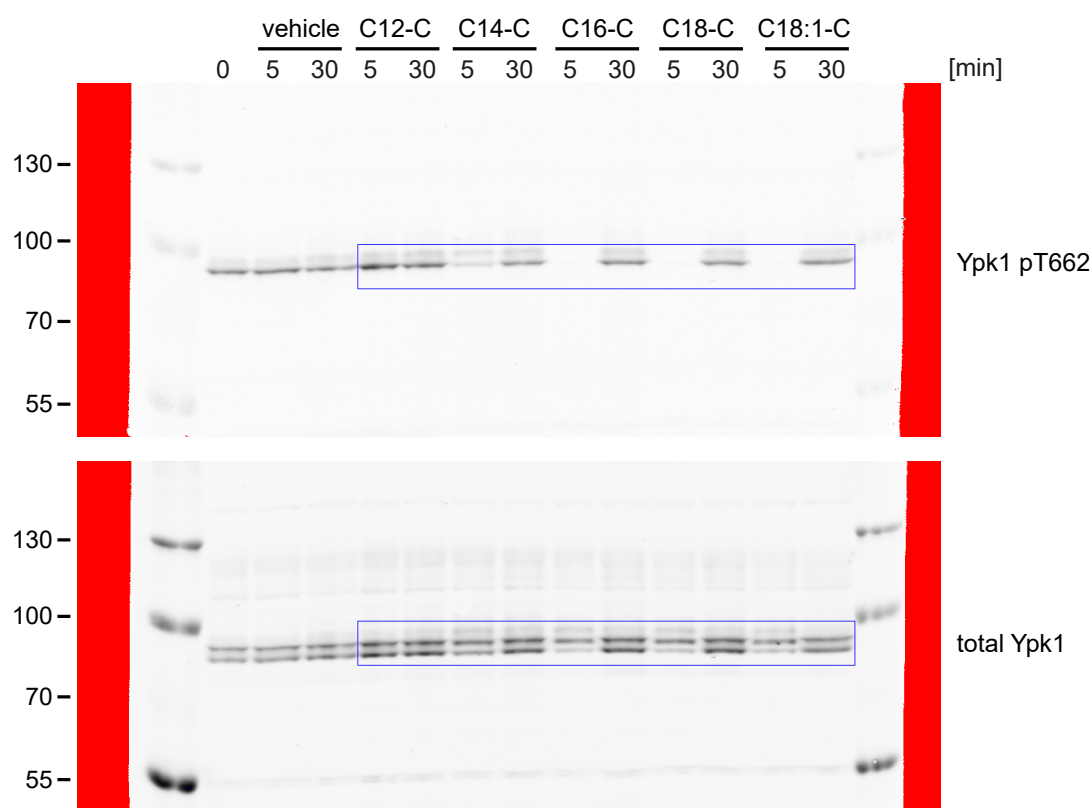

Experiment  
20210825B

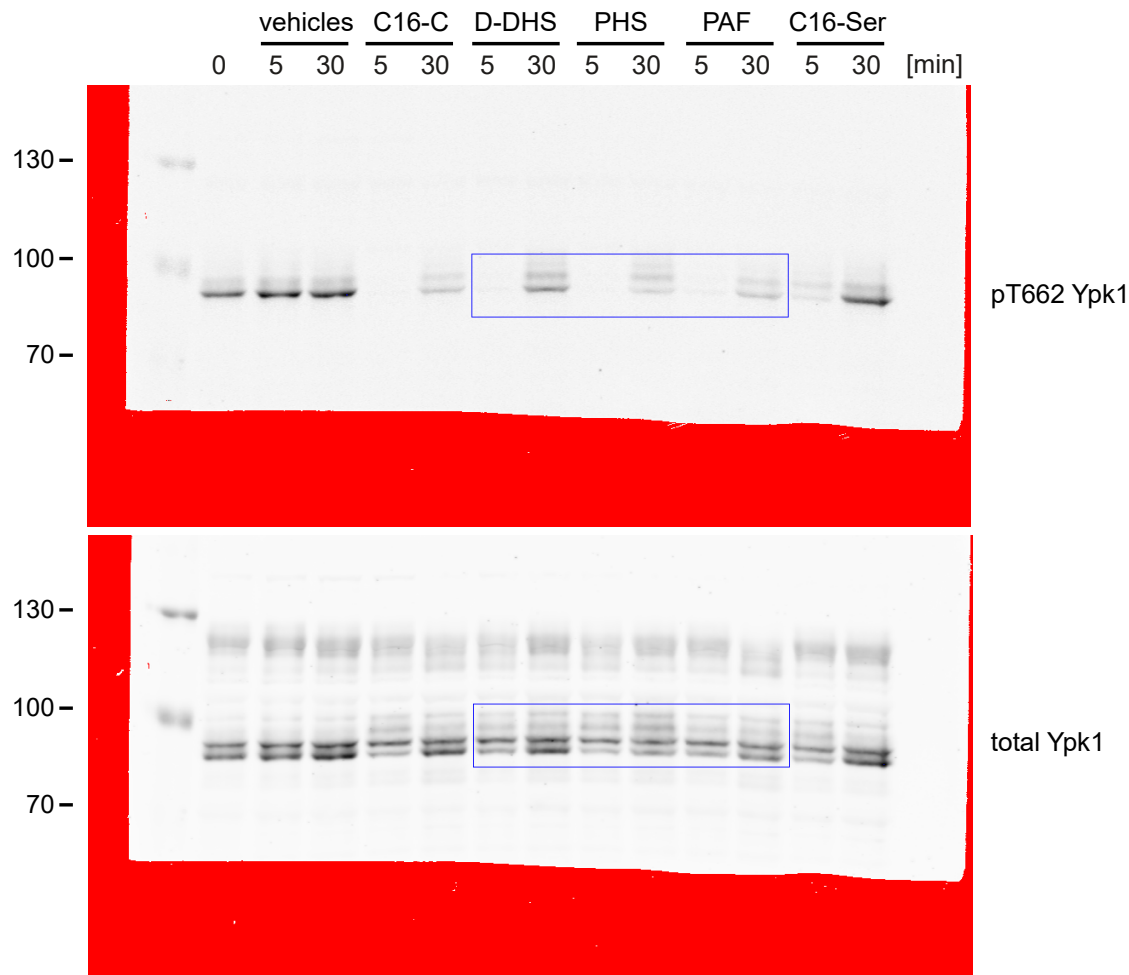

Experiment  
20210915

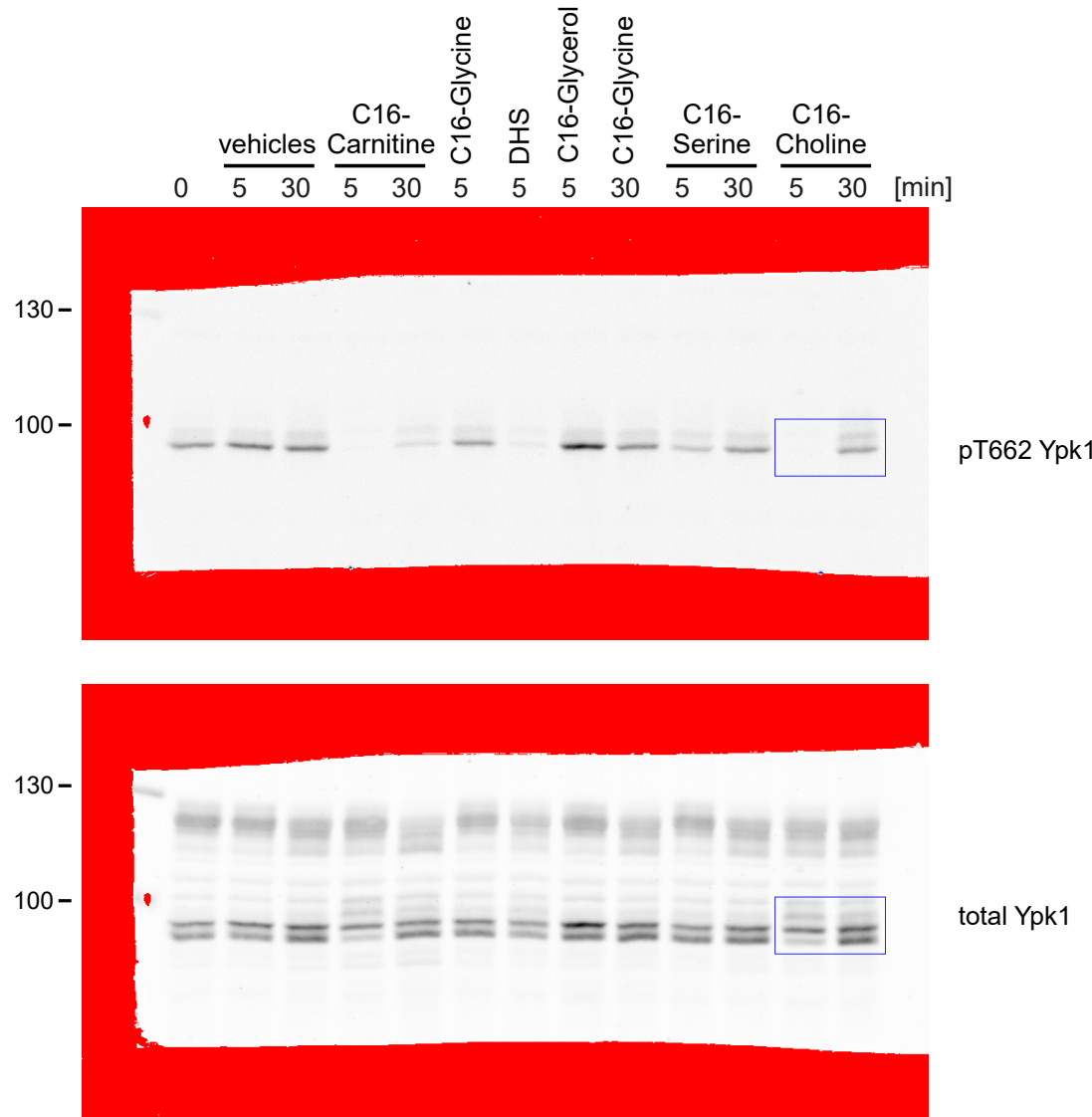

Supplement: Supplementary file 15 — Source data Fig. 1 [file 44318_2025_618_MOESM15_ESM.zip › Figure 1/1D/Uncropped WBs annotated.pdf]

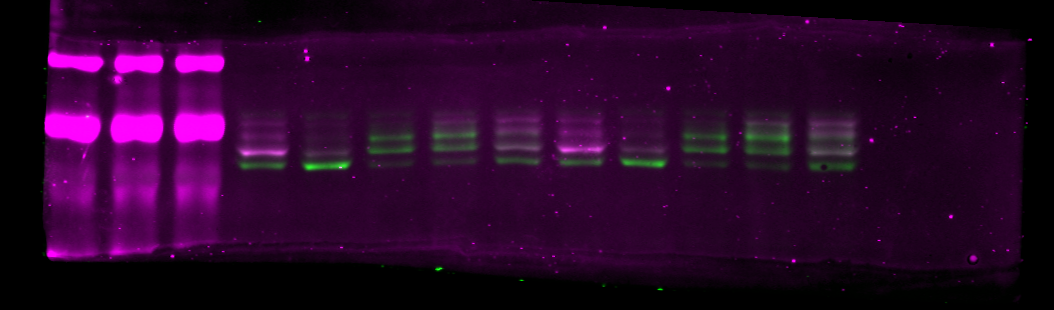

Supplement: Supplementary file 15 — Source data Fig. 1 [file 44318_2025_618_MOESM15_ESM.zip › Figure 1/1E/uncropped WBs/20240530.tif]

**20240530B**

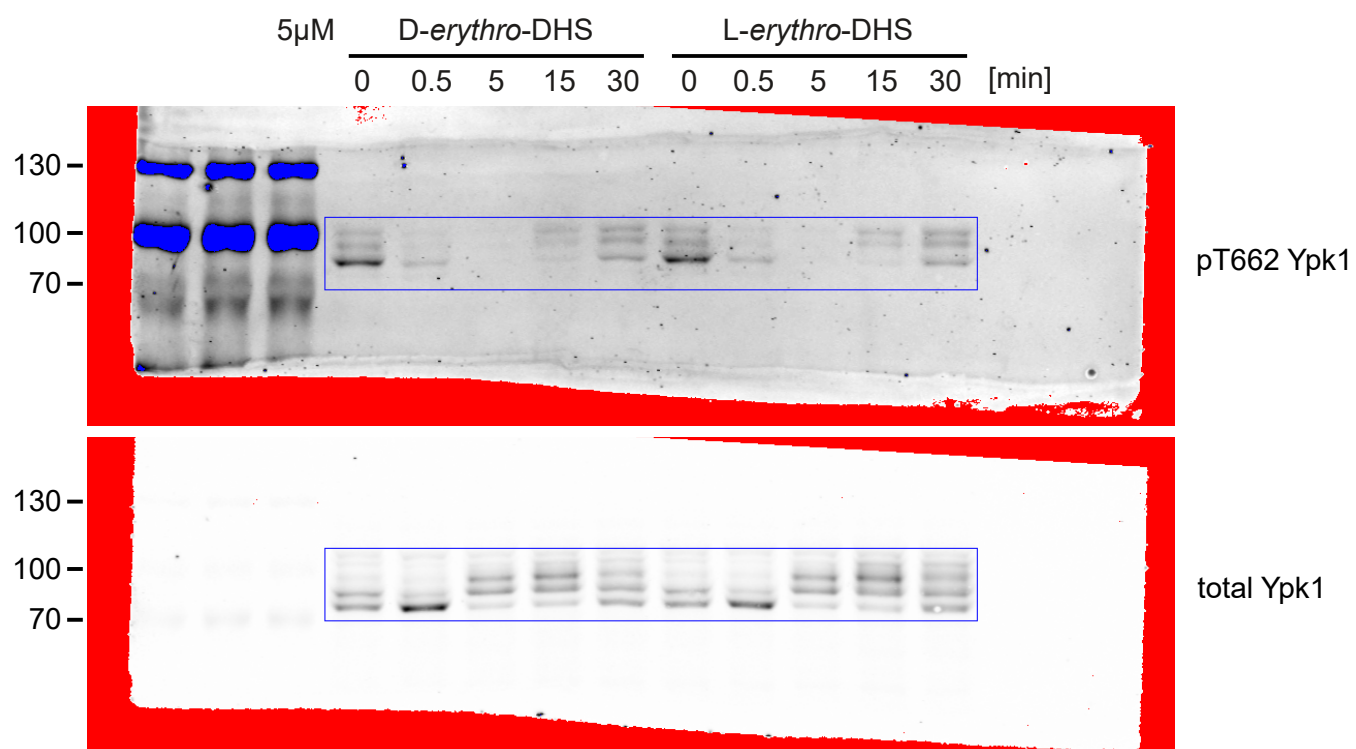

Supplement: Supplementary file 15 — Source data Fig. 1 [file 44318_2025_618_MOESM15_ESM.zip › Figure 1/1E/Uncropped WBs annotated.pdf]

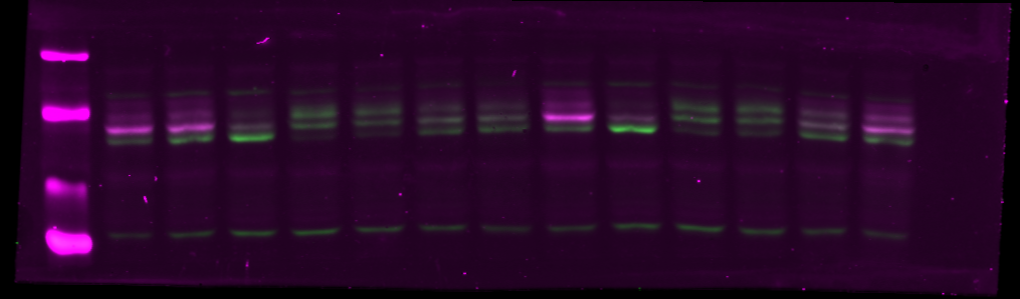

Supplement: Supplementary file 16 — Source data Fig. 2 [file 44318_2025_618_MOESM16_ESM.zip › Figure 2/2D-E/uncropped WBs/20250225B-lamdelta LAMAA_t0.tif]

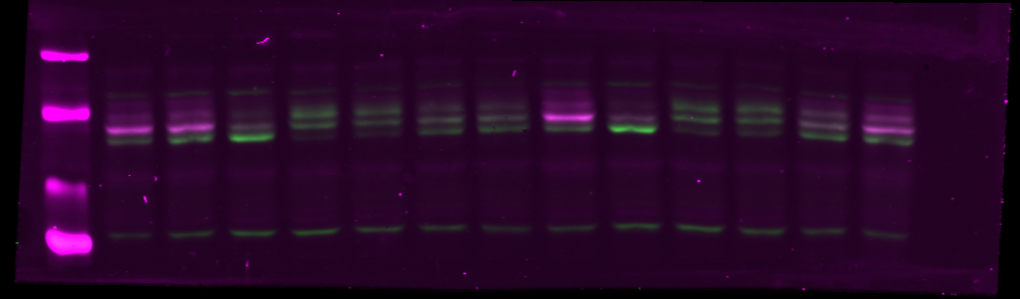

Supplement: Supplementary file 16 — Source data Fig. 2 [file 44318_2025_618_MOESM16_ESM.zip › Figure 2/2D-E/uncropped WBs/20250225B-lamdelta LAMAA_timecourse.tif]

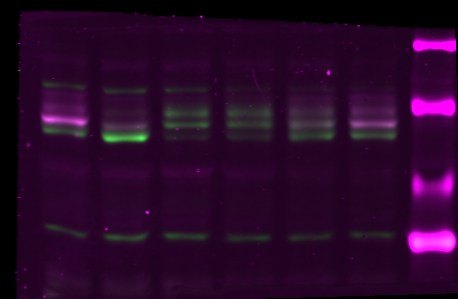

Supplement: Supplementary file 16 — Source data Fig. 2 [file 44318_2025_618_MOESM16_ESM.zip › Figure 2/2D-E/uncropped WBs/20250225B-WT.tif]

**Fig. 2D: Experiment 20250225B**

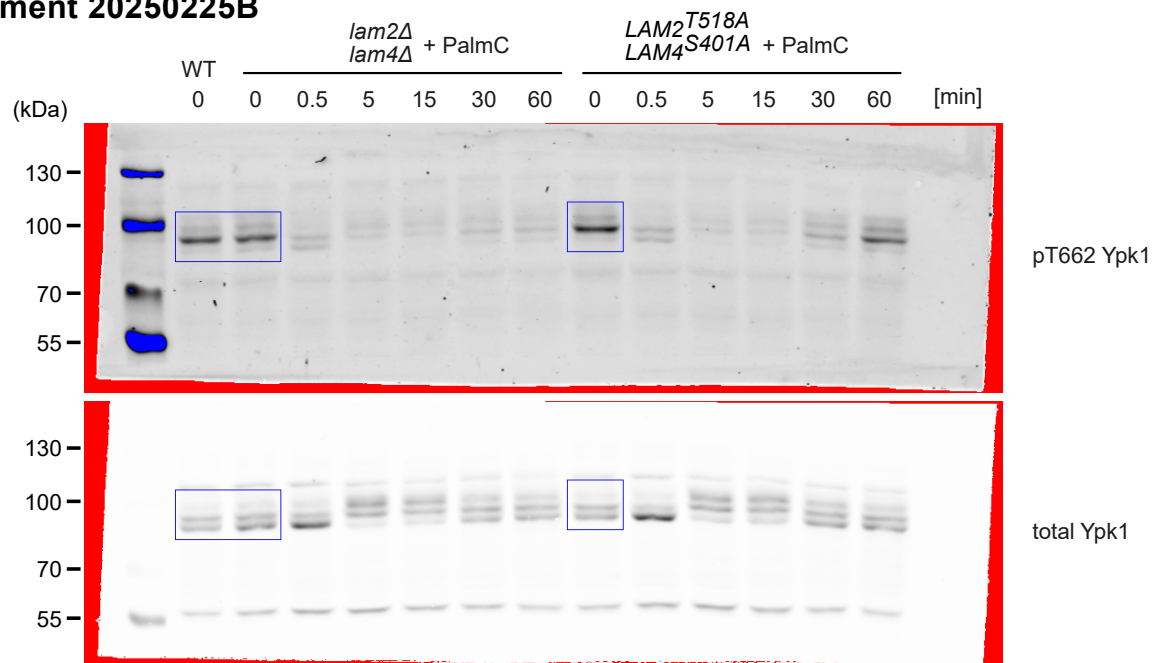

**Fig. 2E: Experiment 20250225B**

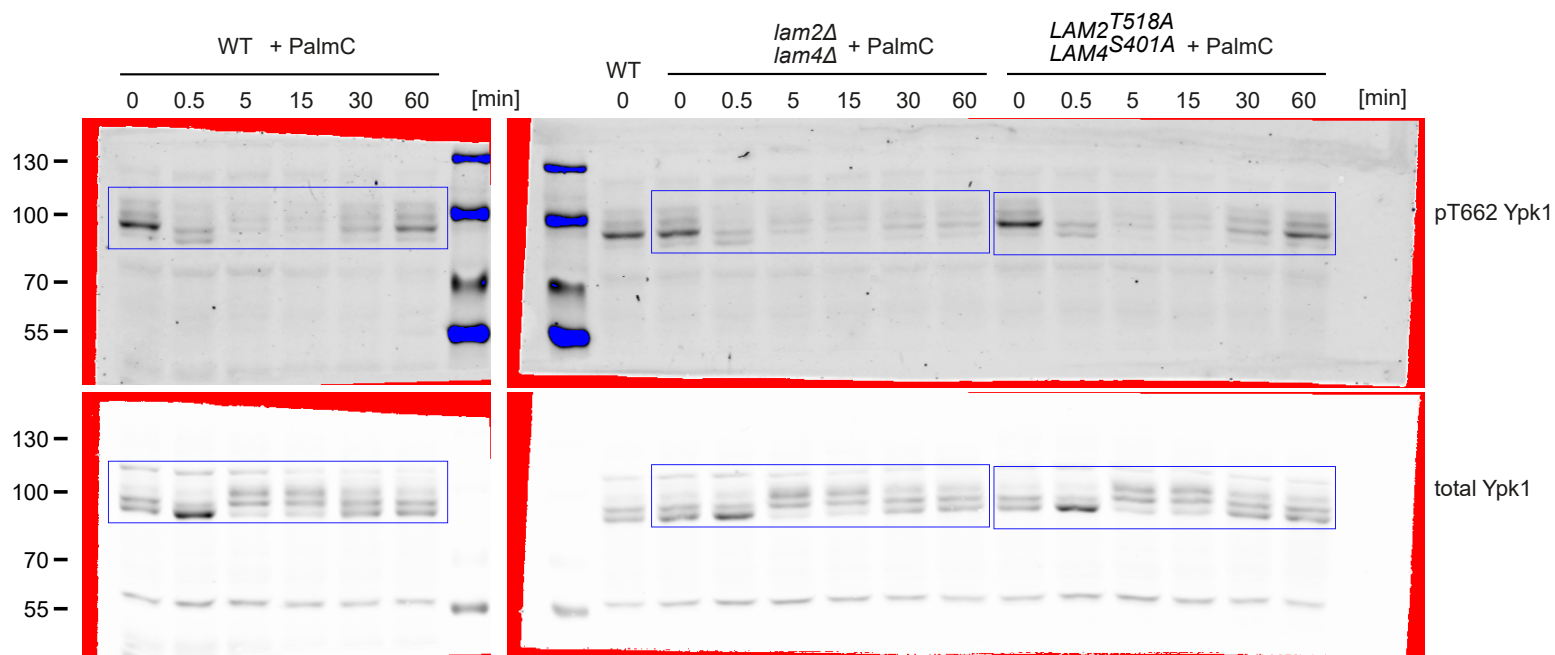

Supplement: Supplementary file 16 — Source data Fig. 2 [file 44318_2025_618_MOESM16_ESM.zip › Figure 2/2D-E/Uncropped WBs annotated.pdf]

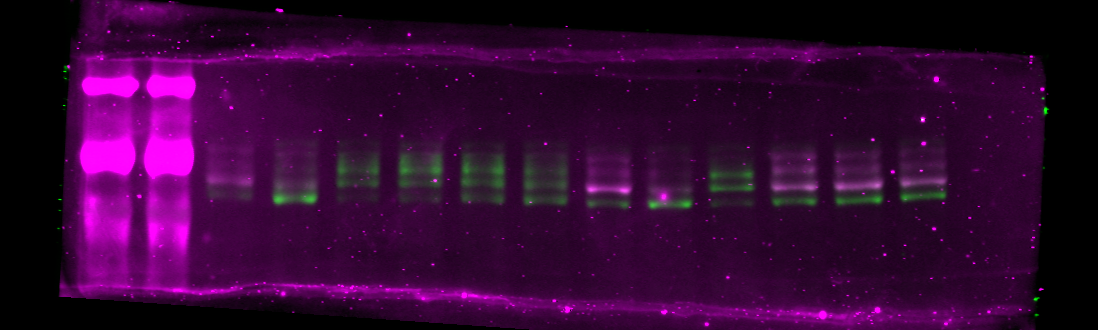

Supplement: Supplementary file 19 — EV Figures Source Data [file 44318_2025_618_MOESM19_ESM.zip › Expanded View Figures/Figure EV1/EV1A/uncropped WB images/20240531.tif]

## Experiment 20240531

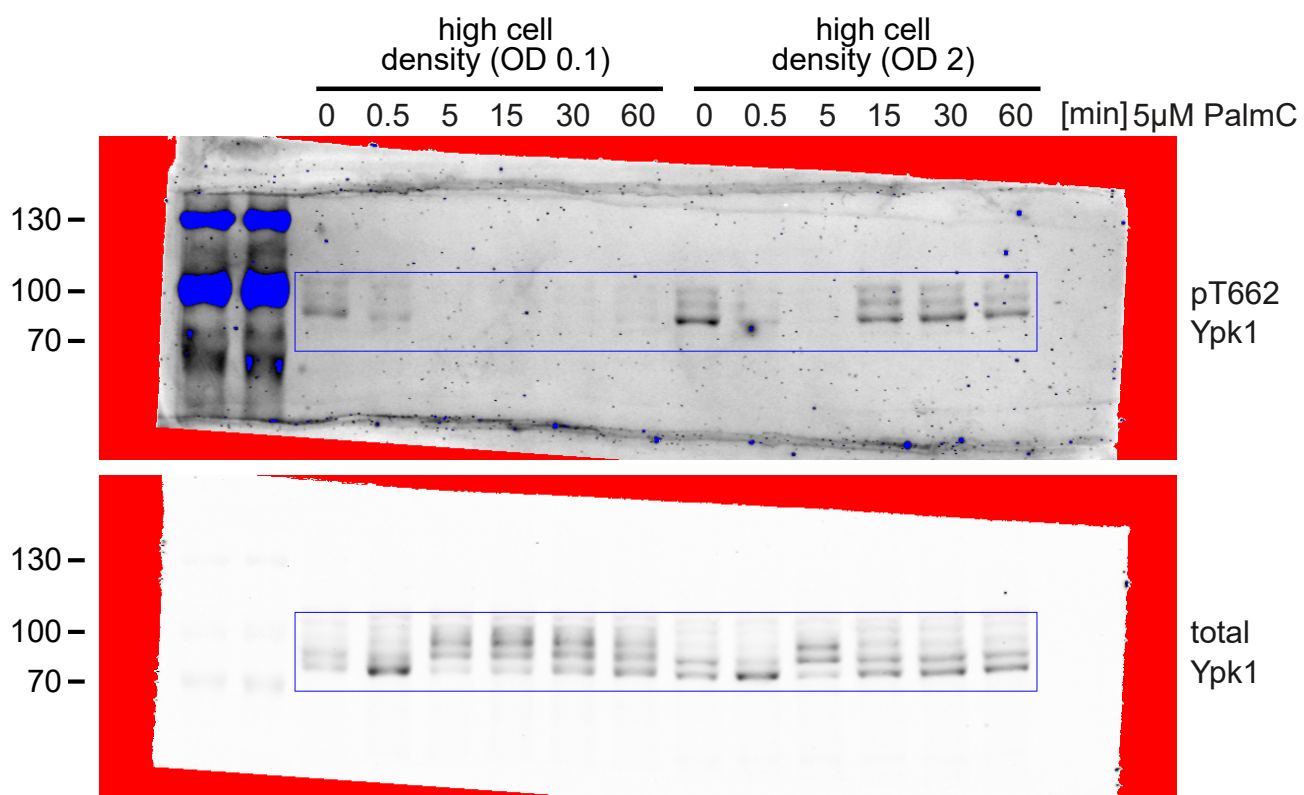

Supplement: Supplementary file 19 — EV Figures Source Data [file 44318_2025_618_MOESM19_ESM.zip › Expanded View Figures/Figure EV1/EV1A/Uncropped WBs annotated.pdf]

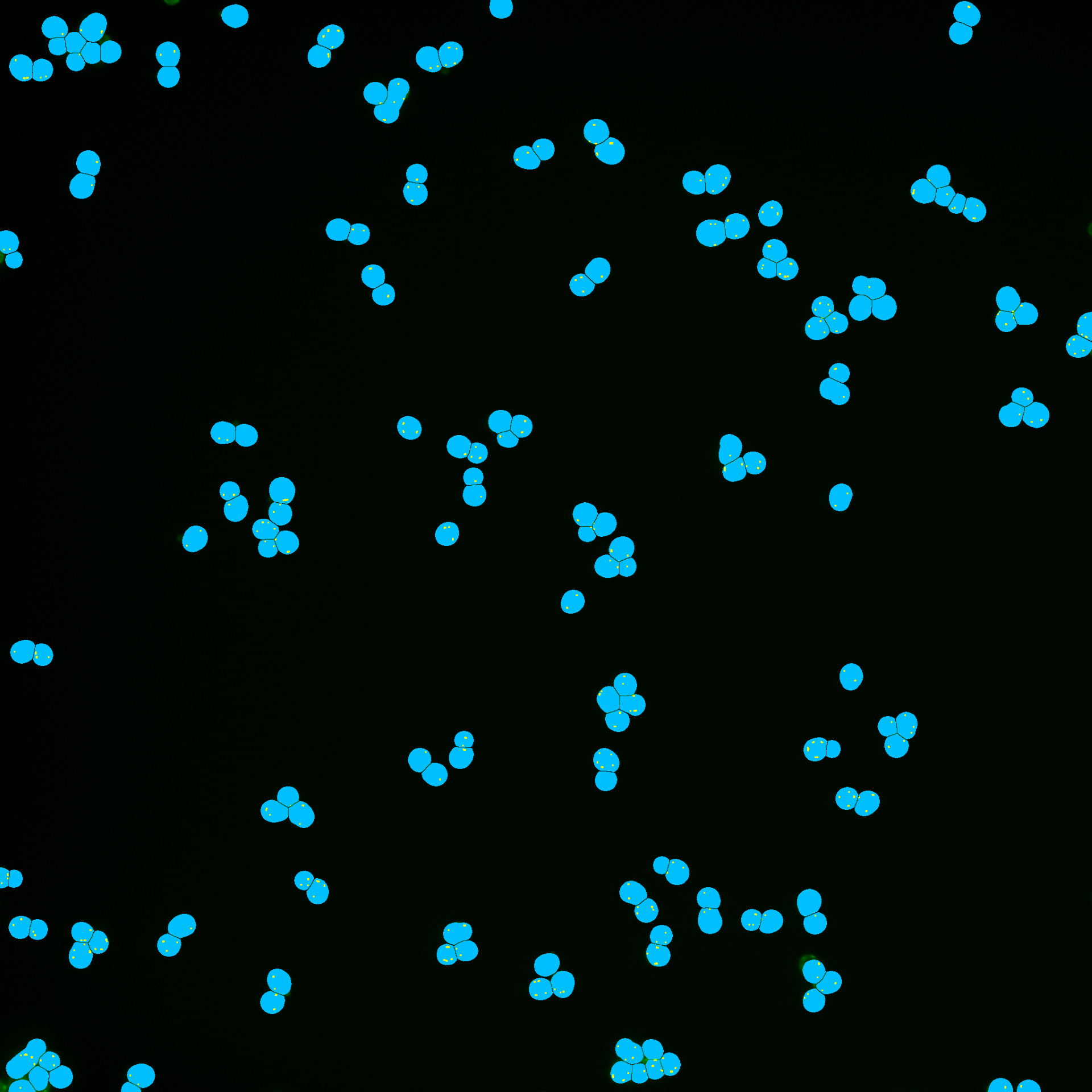

Supplement: Supplementary file 19 — EV Figures Source Data [file 44318_2025_618_MOESM19_ESM.zip › Expanded View Figures/Figure EV1/EV1B/PalmC segmented.tif]

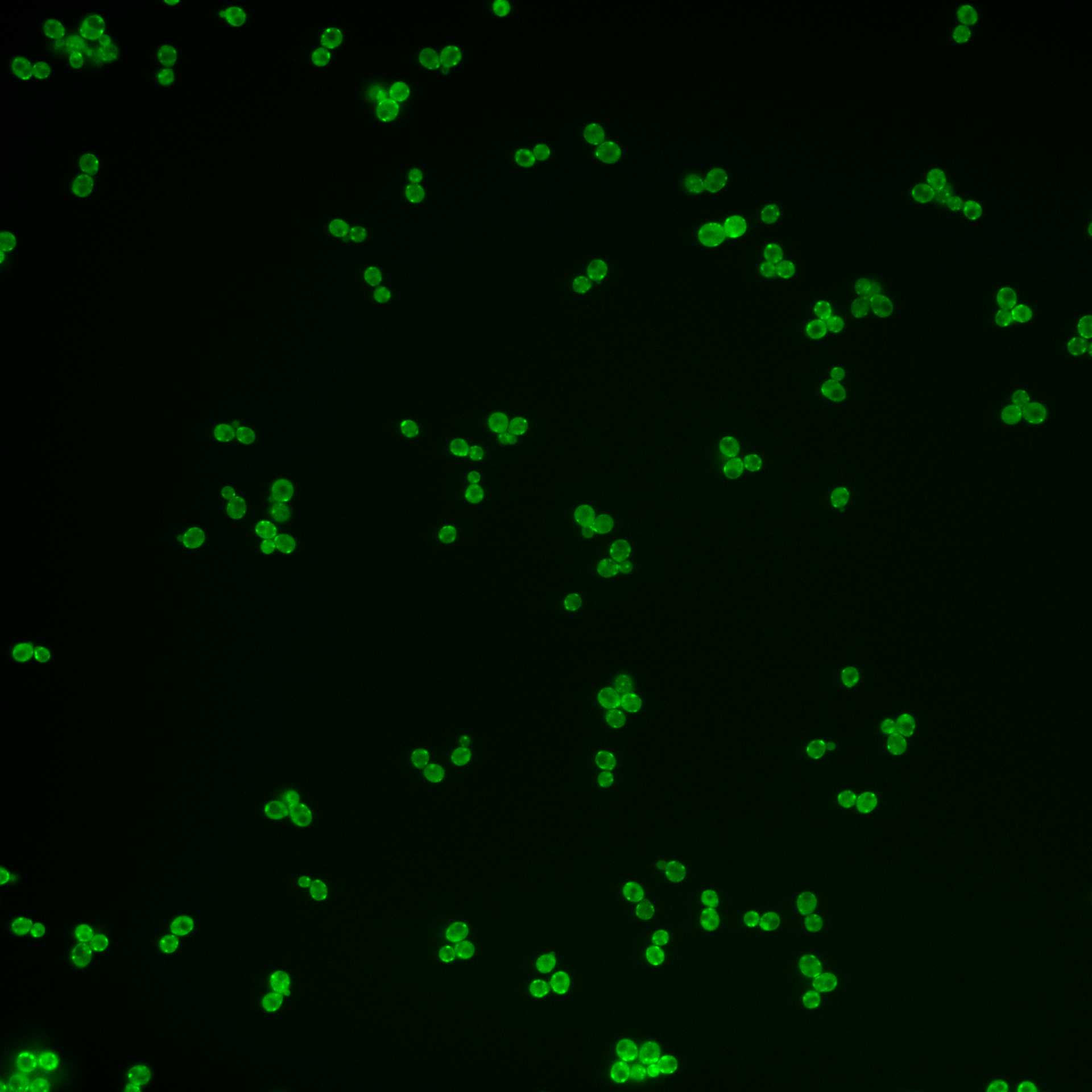

Supplement: Supplementary file 19 — EV Figures Source Data [file 44318_2025_618_MOESM19_ESM.zip › Expanded View Figures/Figure EV1/EV1B/PalmC.tif]

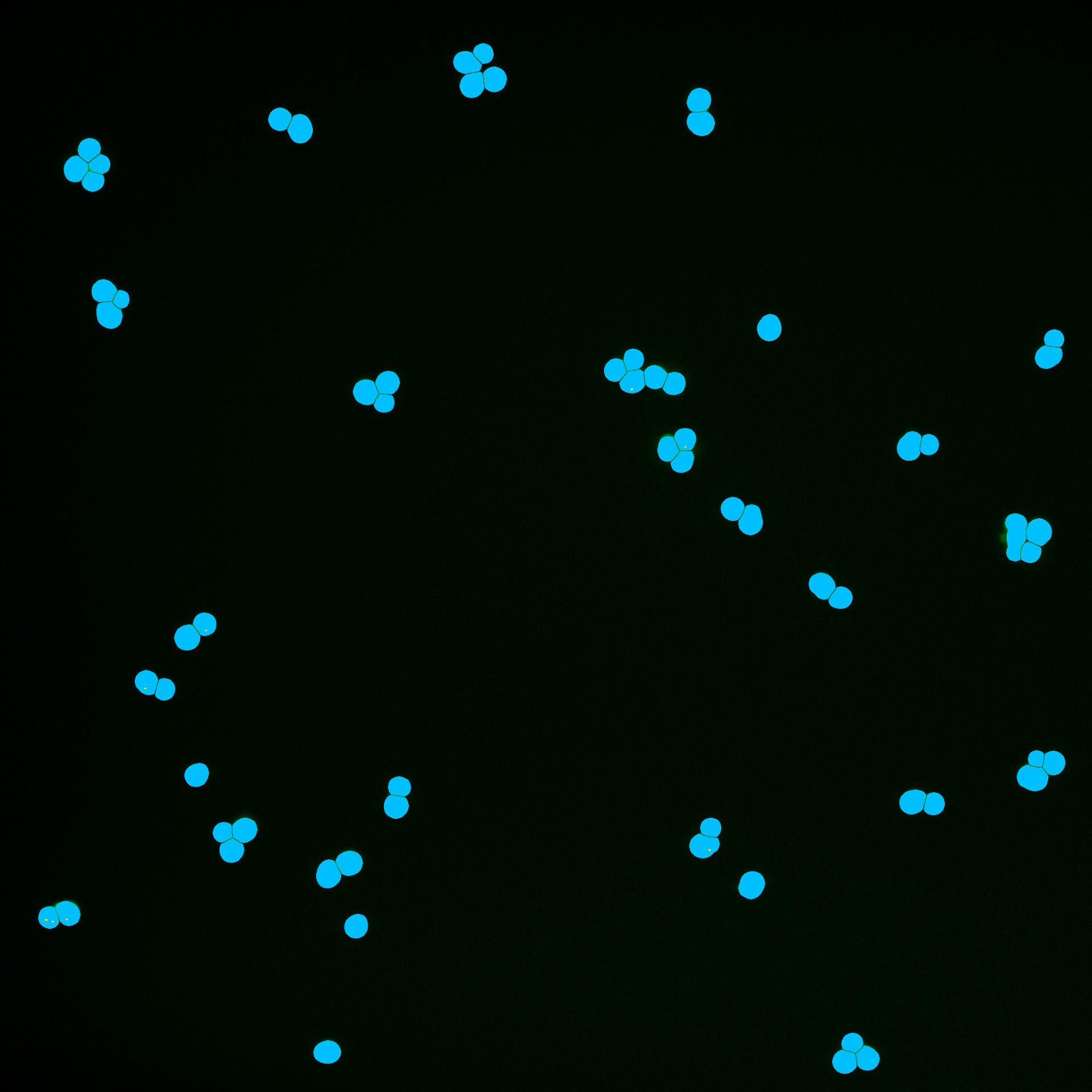

Supplement: Supplementary file 19 — EV Figures Source Data [file 44318_2025_618_MOESM19_ESM.zip › Expanded View Figures/Figure EV1/EV1B/untreated segmented.tif]

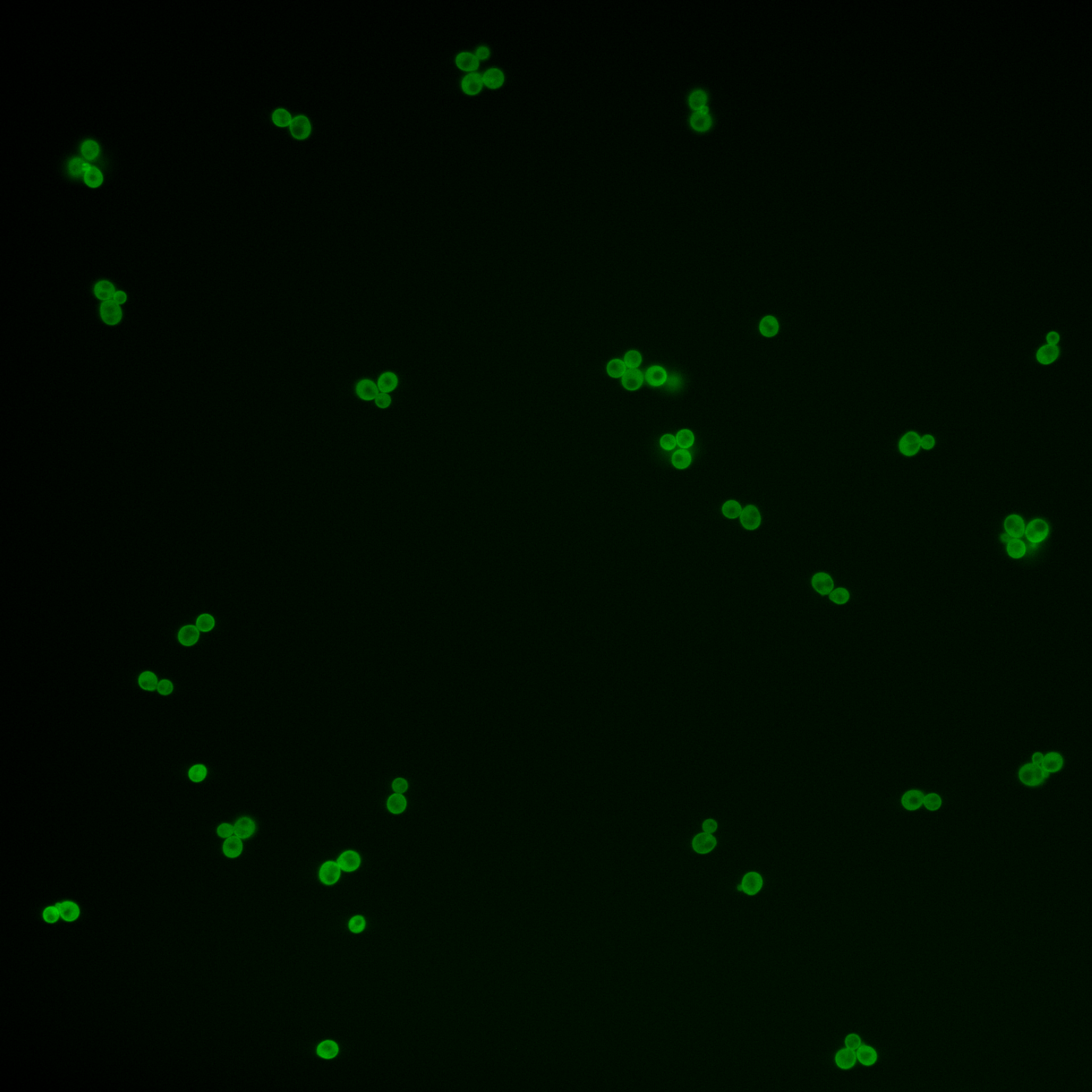

Supplement: Supplementary file 19 — EV Figures Source Data [file 44318_2025_618_MOESM19_ESM.zip › Expanded View Figures/Figure EV1/EV1B/untreated.tif]

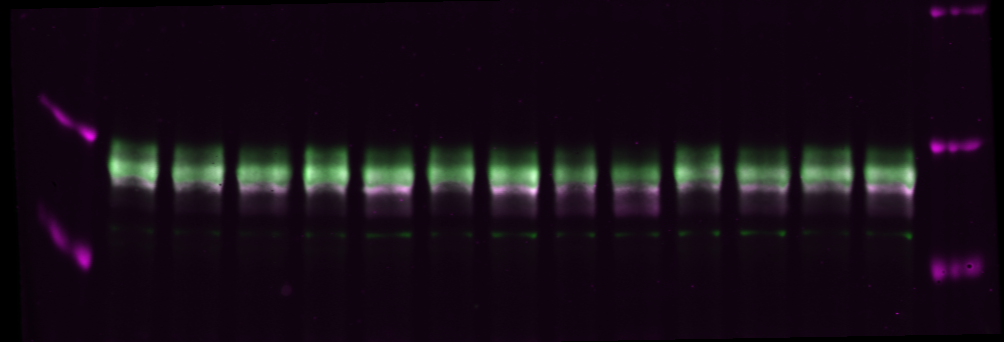

Supplement: Supplementary file 19 — EV Figures Source Data [file 44318_2025_618_MOESM19_ESM.zip › Expanded View Figures/Figure EV1/EV1C/uncropped WBs/20210804B.tif]

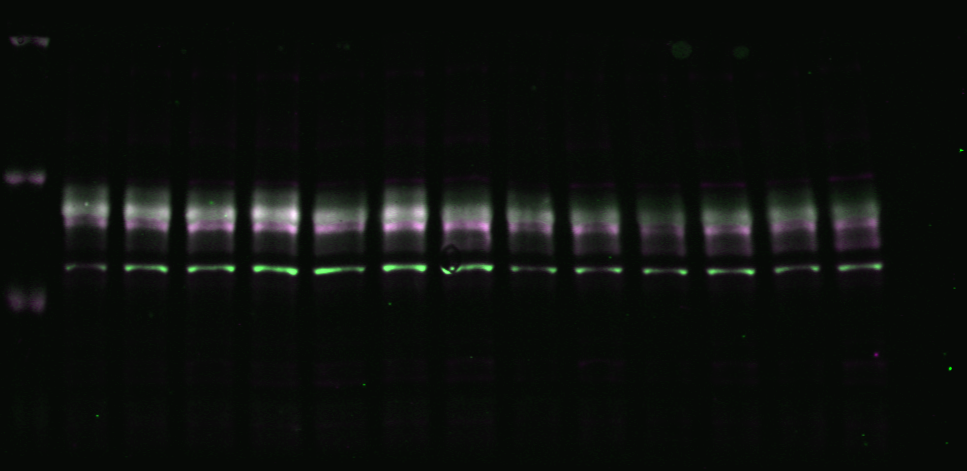

Supplement: Supplementary file 19 — EV Figures Source Data [file 44318_2025_618_MOESM19_ESM.zip › Expanded View Figures/Figure EV1/EV1C/uncropped WBs/20210806.tif]

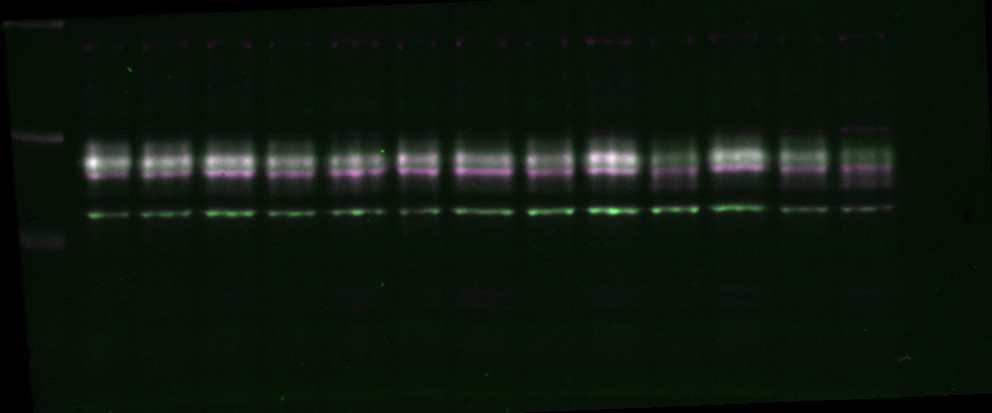

Supplement: Supplementary file 19 — EV Figures Source Data [file 44318_2025_618_MOESM19_ESM.zip › Expanded View Figures/Figure EV1/EV1C/uncropped WBs/20210820.tif]

experiment 20210804B

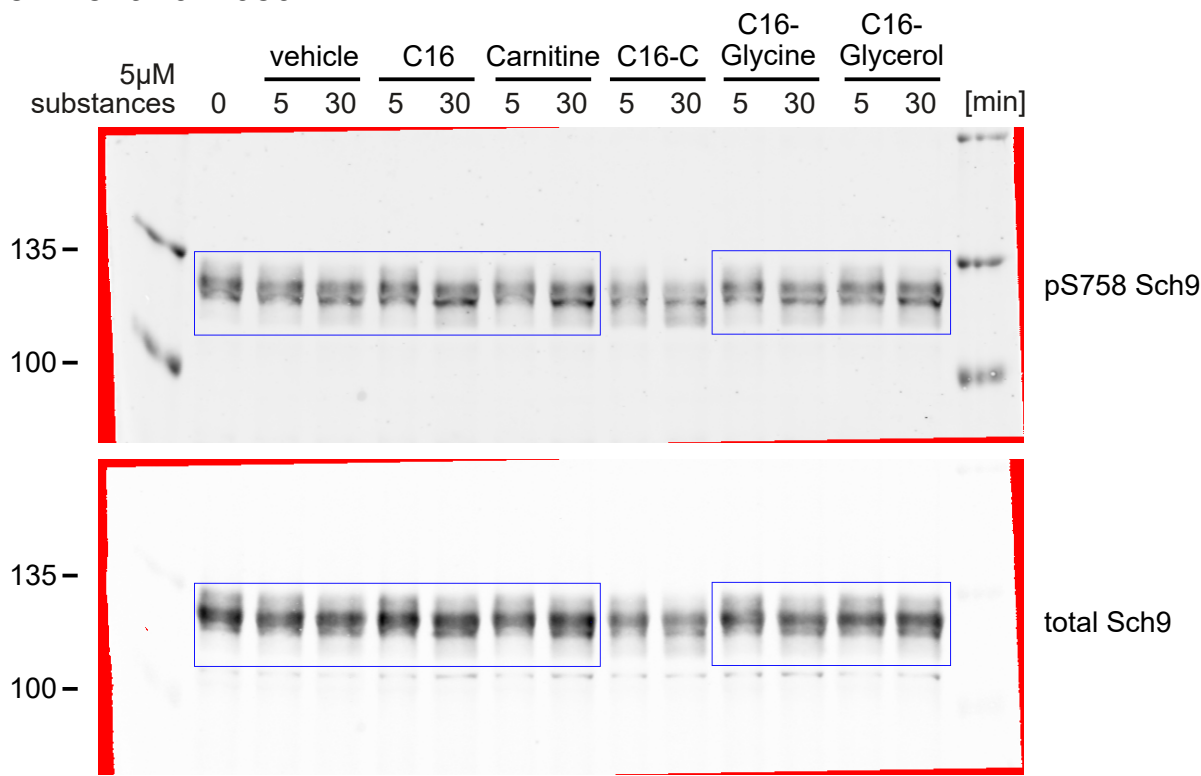

experiment 20210820

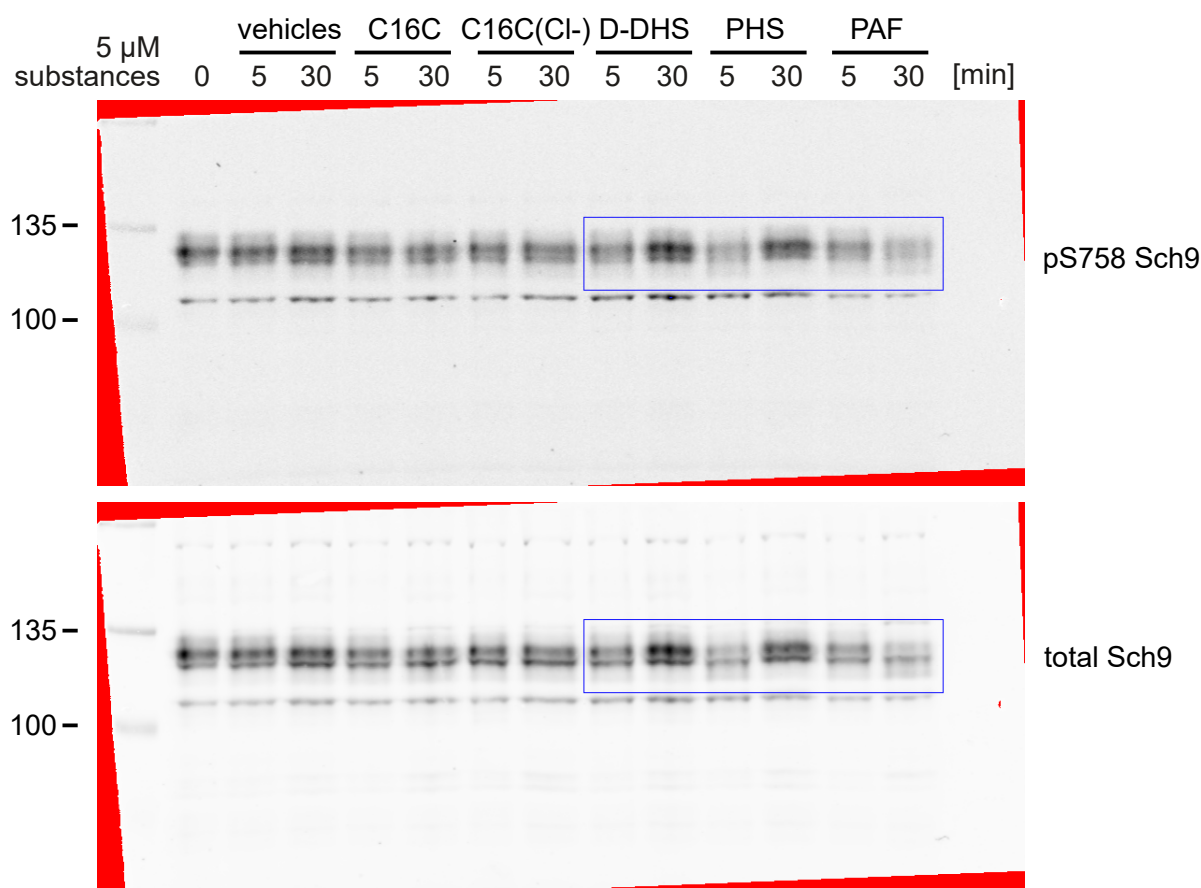

20210806

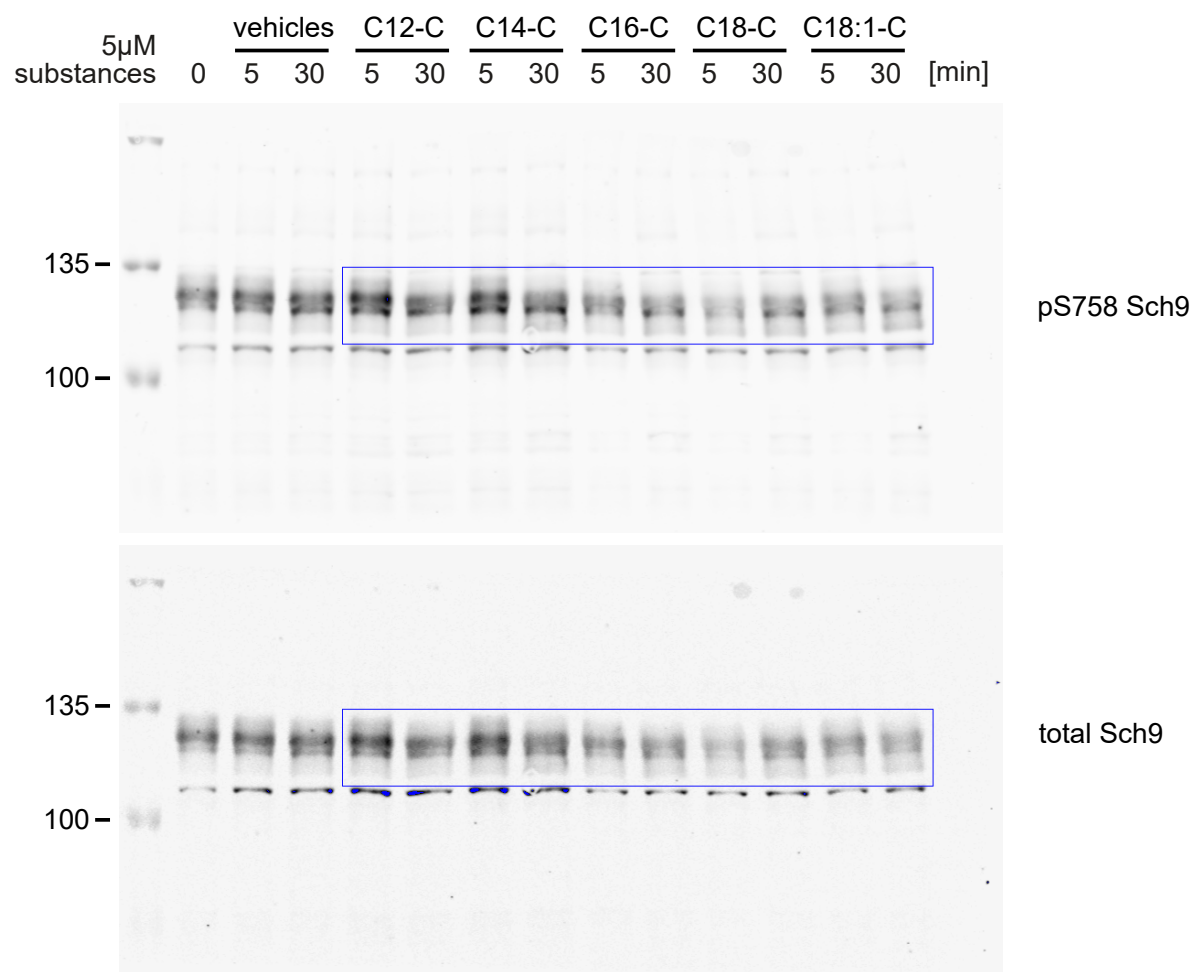

Supplement: Supplementary file 19 — EV Figures Source Data [file 44318_2025_618_MOESM19_ESM.zip › Expanded View Figures/Figure EV1/EV1C/Uncropped WBs annotated.pdf]

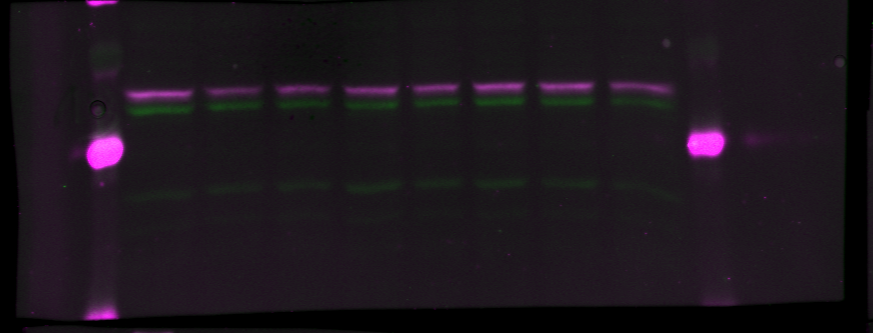

Supplement: Supplementary file 19 — EV Figures Source Data [file 44318_2025_618_MOESM19_ESM.zip › Expanded View Figures/Figure EV1/EV1D/uncropped WBs/17-08-2020-1.tif]

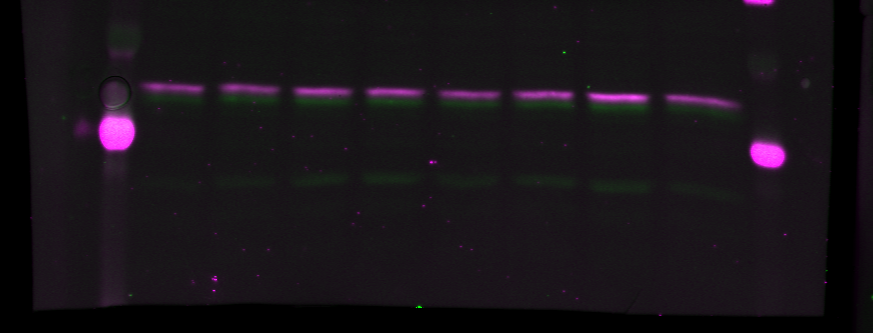

Supplement: Supplementary file 19 — EV Figures Source Data [file 44318_2025_618_MOESM19_ESM.zip › Expanded View Figures/Figure EV1/EV1D/uncropped WBs/17-08-2020-3.tif]

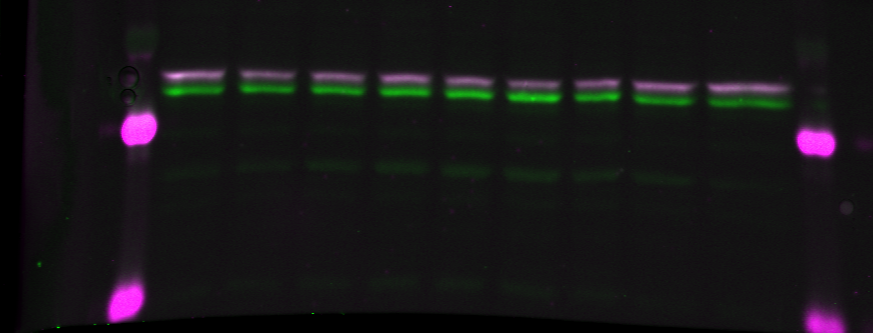

Supplement: Supplementary file 19 — EV Figures Source Data [file 44318_2025_618_MOESM19_ESM.zip › Expanded View Figures/Figure EV1/EV1D/uncropped WBs/17-08-2020-4.tif]

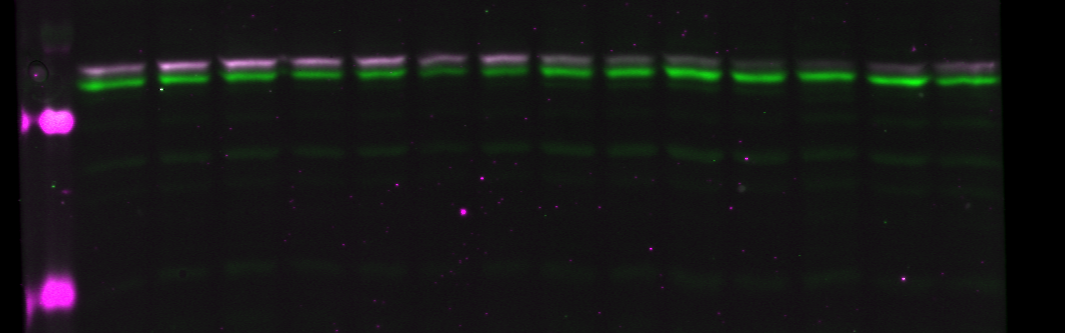

Supplement: Supplementary file 19 — EV Figures Source Data [file 44318_2025_618_MOESM19_ESM.zip › Expanded View Figures/Figure EV1/EV1D/uncropped WBs/17-08-2020-5.tif]

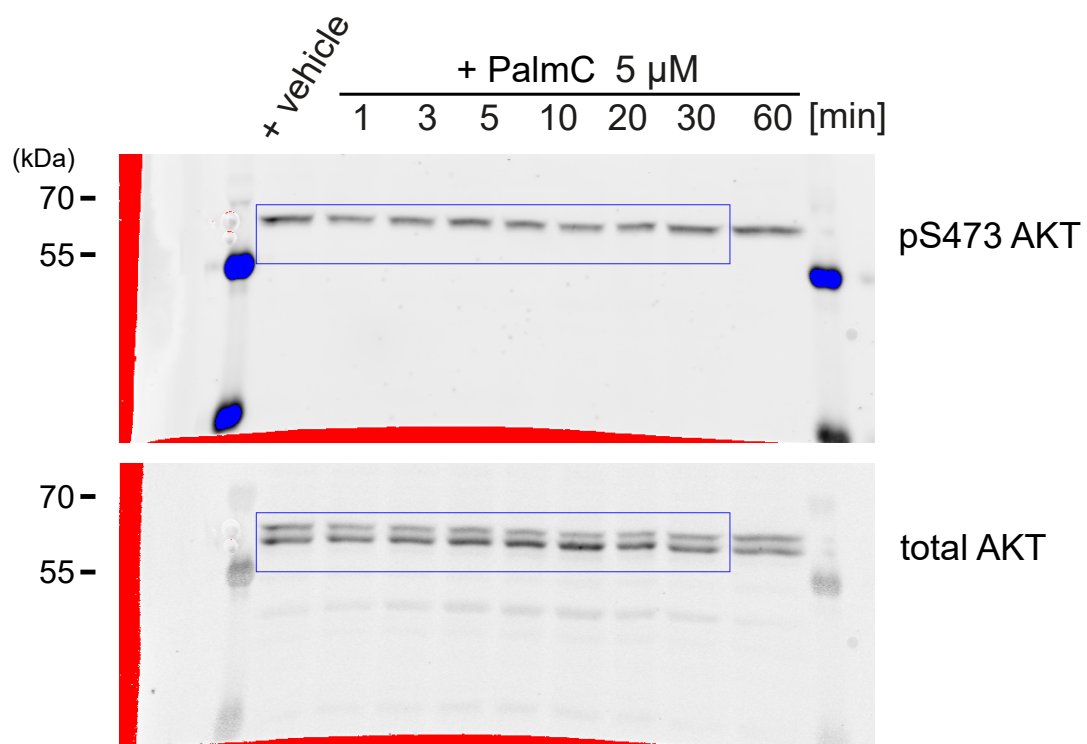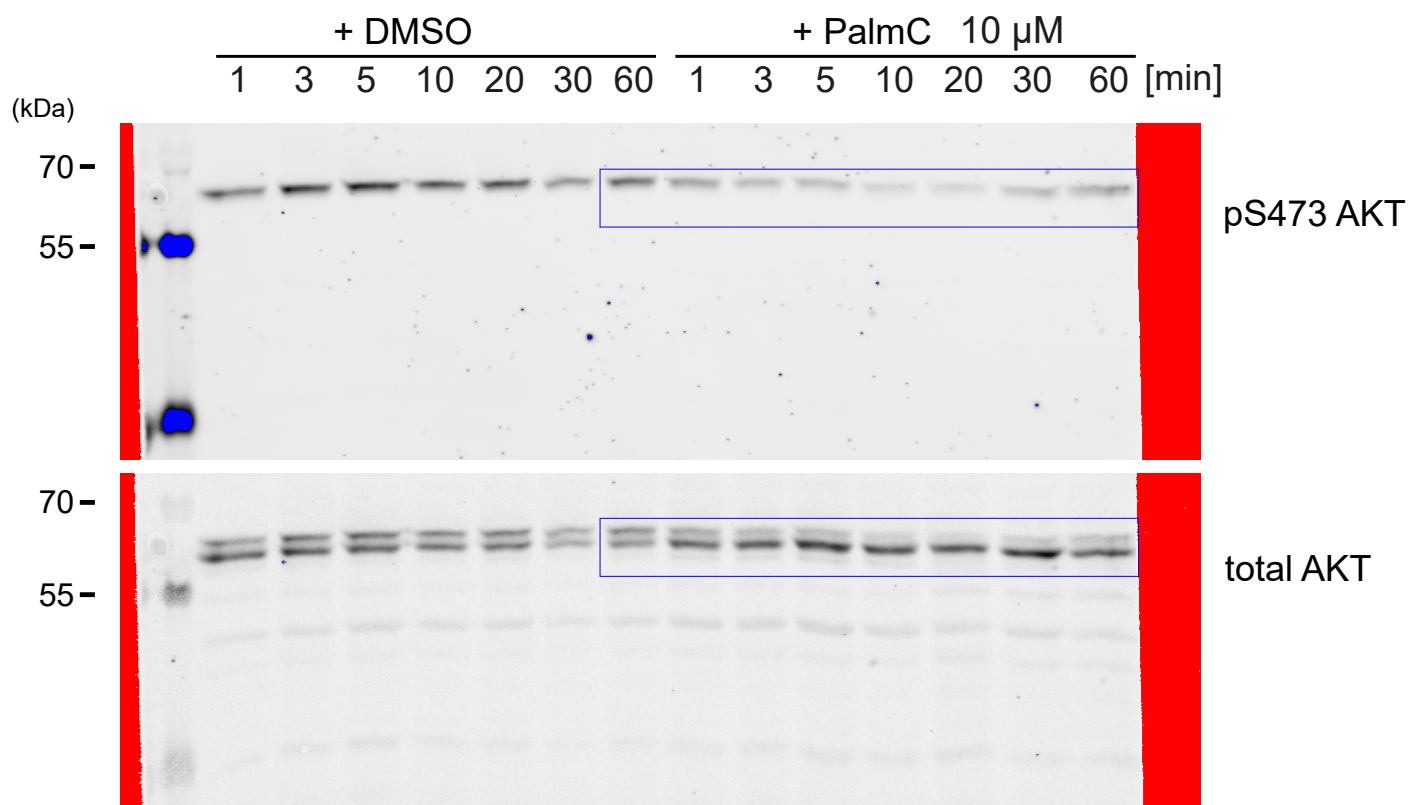

Supplement: Supplementary file 19 — EV Figures Source Data [file 44318_2025_618_MOESM19_ESM.zip › Expanded View Figures/Figure EV1/EV1D/Uncropped WBs annotated.pdf]

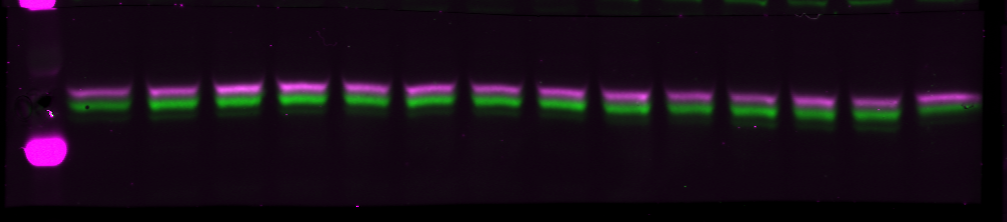

Supplement: Supplementary file 19 — EV Figures Source Data [file 44318_2025_618_MOESM19_ESM.zip › Expanded View Figures/Figure EV1/EV1E/uncropped WB images/WB 1 unc.tif]

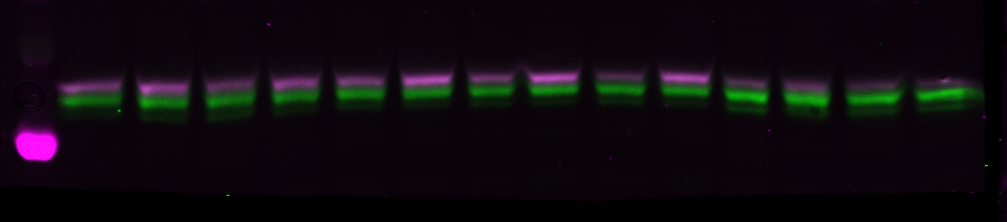

Supplement: Supplementary file 19 — EV Figures Source Data [file 44318_2025_618_MOESM19_ESM.zip › Expanded View Figures/Figure EV1/EV1E/uncropped WB images/WB 2 unc.tif]

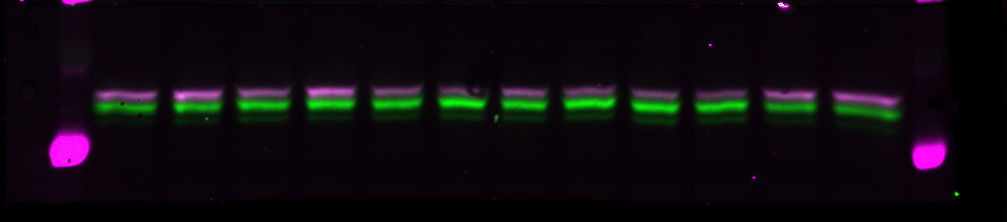

Supplement: Supplementary file 19 — EV Figures Source Data [file 44318_2025_618_MOESM19_ESM.zip › Expanded View Figures/Figure EV1/EV1E/uncropped WB images/WB 3 unc.tif]

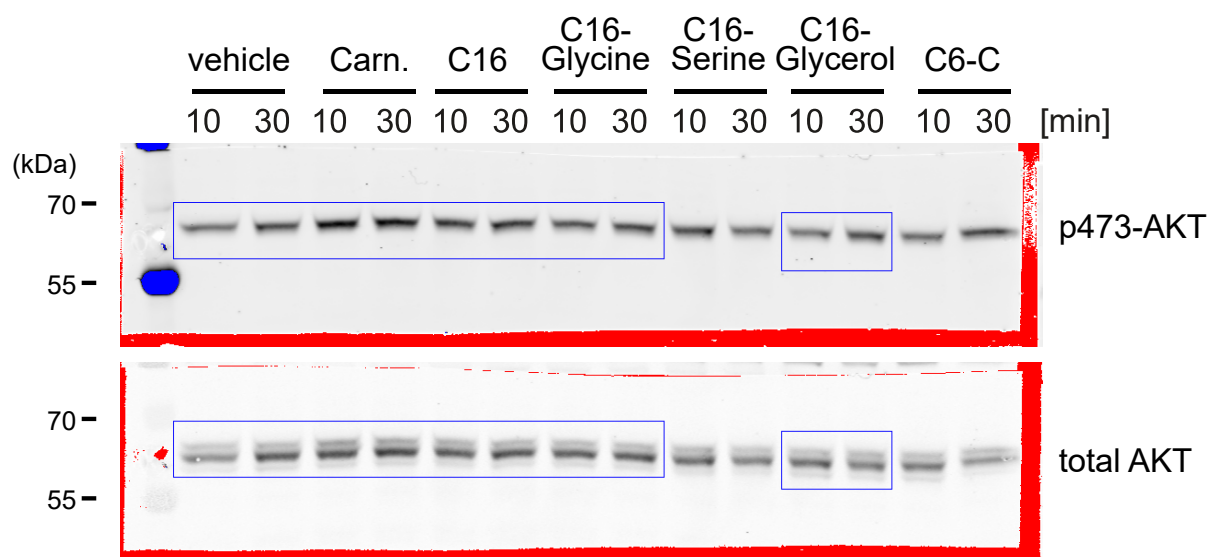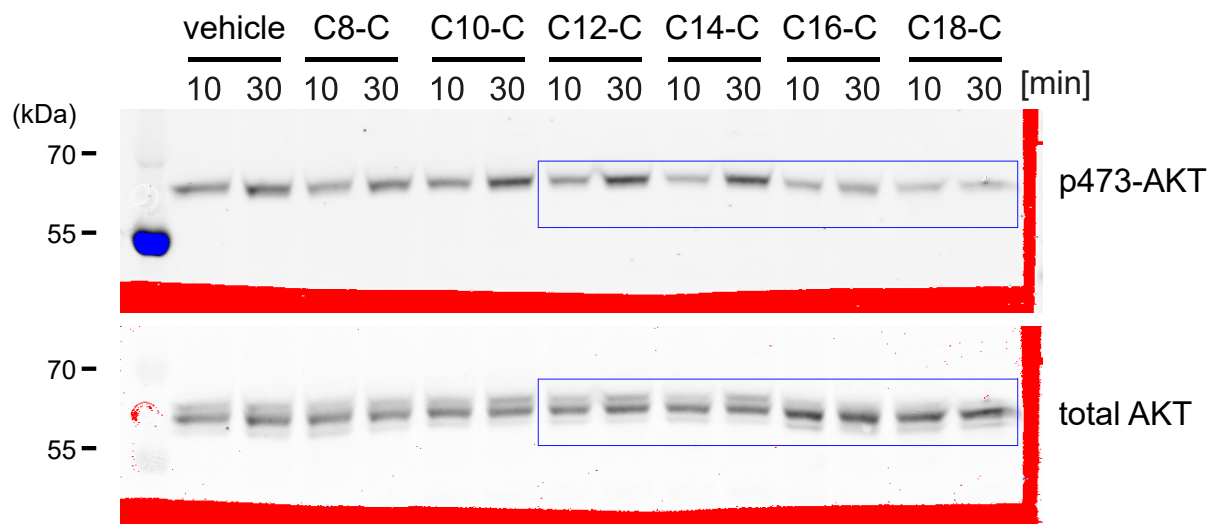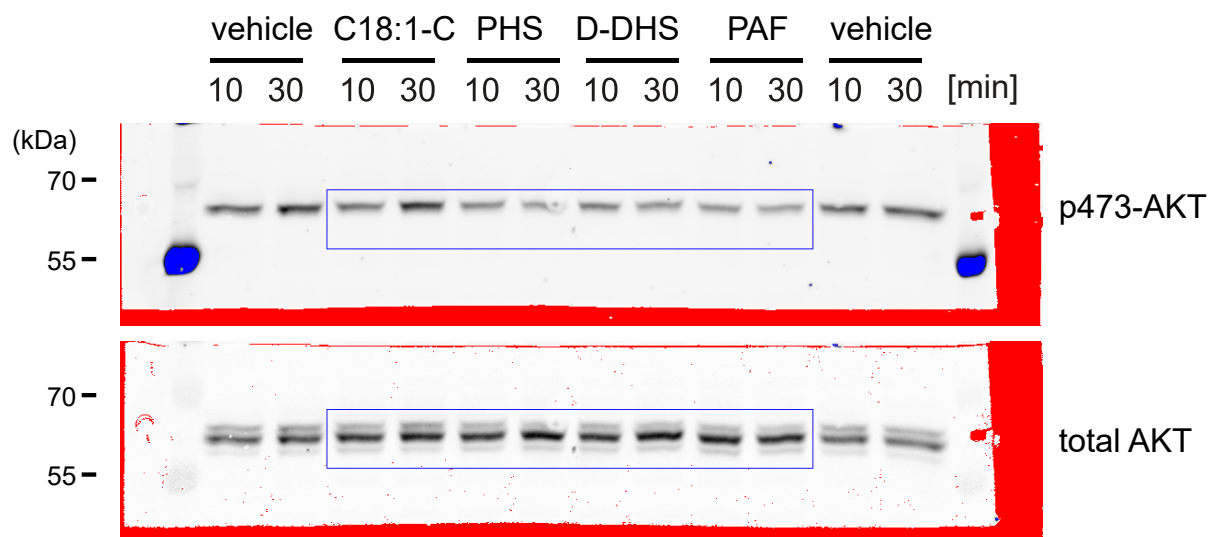

Supplement: Supplementary file 19 — EV Figures Source Data [file 44318_2025_618_MOESM19_ESM.zip › Expanded View Figures/Figure EV1/EV1E/uncropped WBs annotated.pdf]

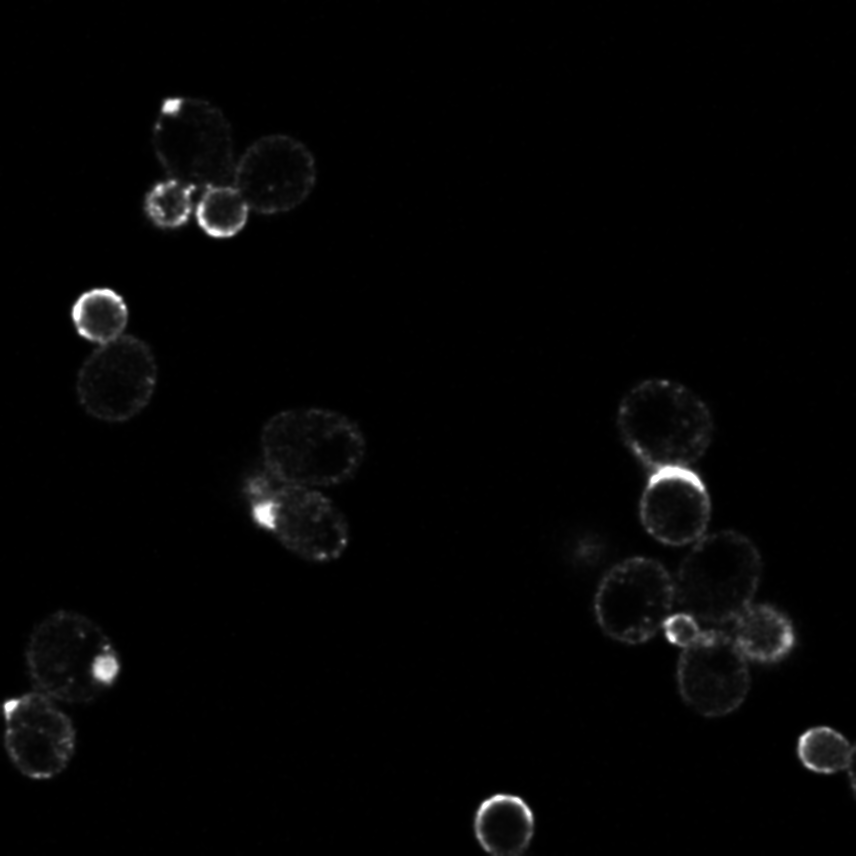

Supplement: Supplementary file 19 — EV Figures Source Data [file 44318_2025_618_MOESM19_ESM.zip › Expanded View Figures/Figure EV2/EV2E/GFP-D4H dsRED-HDEL t0.tif]

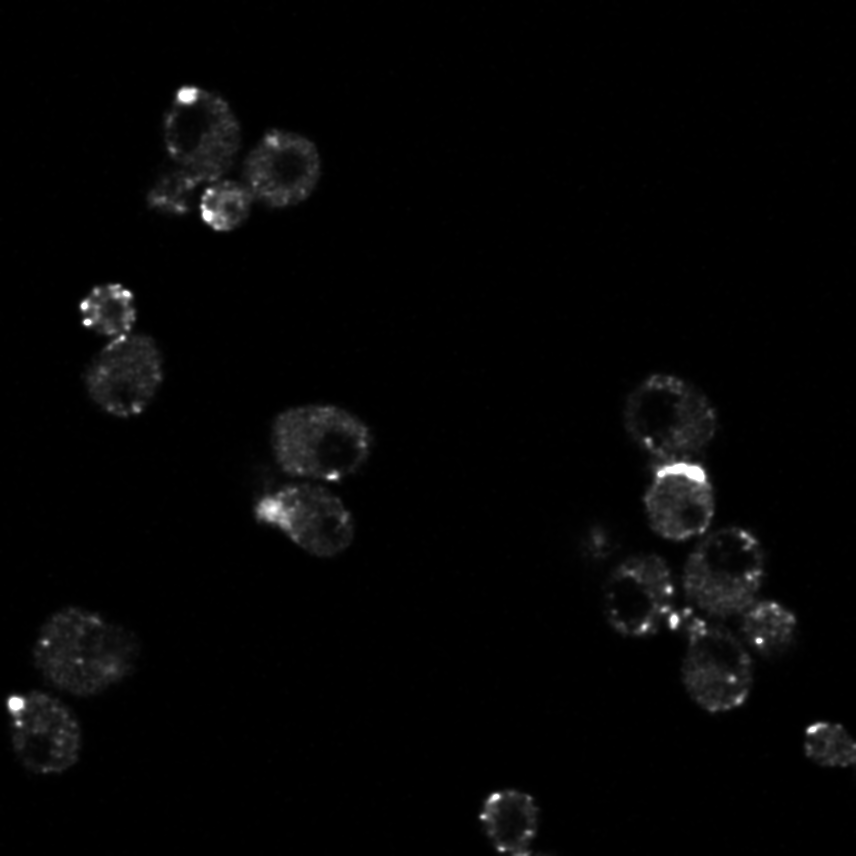

Supplement: Supplementary file 19 — EV Figures Source Data [file 44318_2025_618_MOESM19_ESM.zip › Expanded View Figures/Figure EV2/EV2E/GFP-D4H dsRED-HDEL t1.tif]

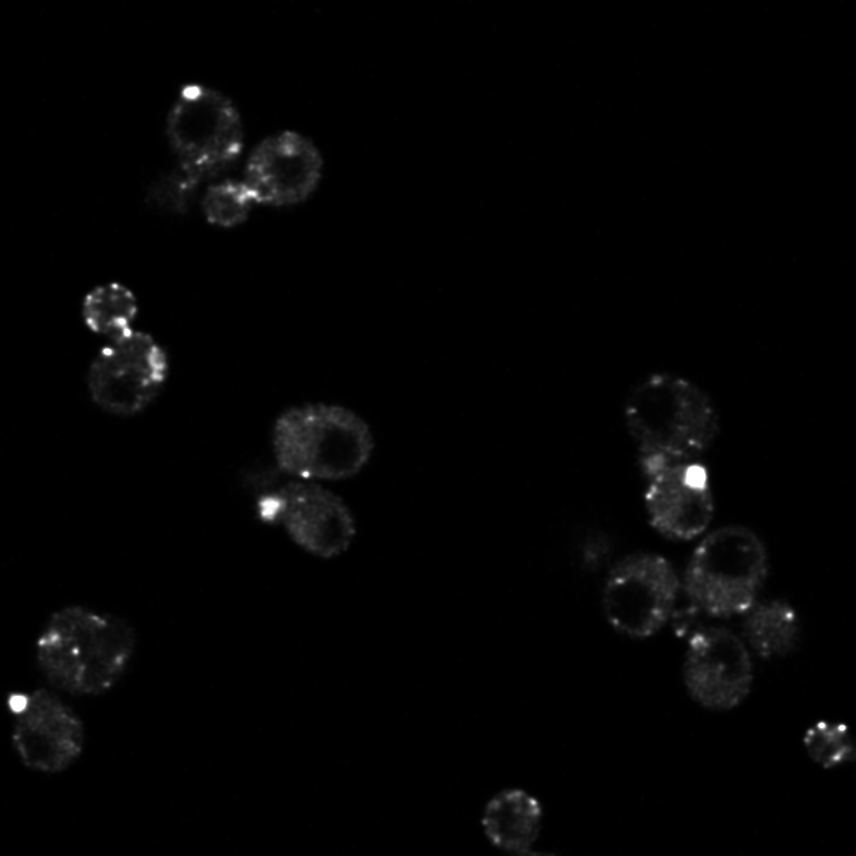

Supplement: Supplementary file 19 — EV Figures Source Data [file 44318_2025_618_MOESM19_ESM.zip › Expanded View Figures/Figure EV2/EV2E/GFP-D4H dsRED-HDEL t2.tif]

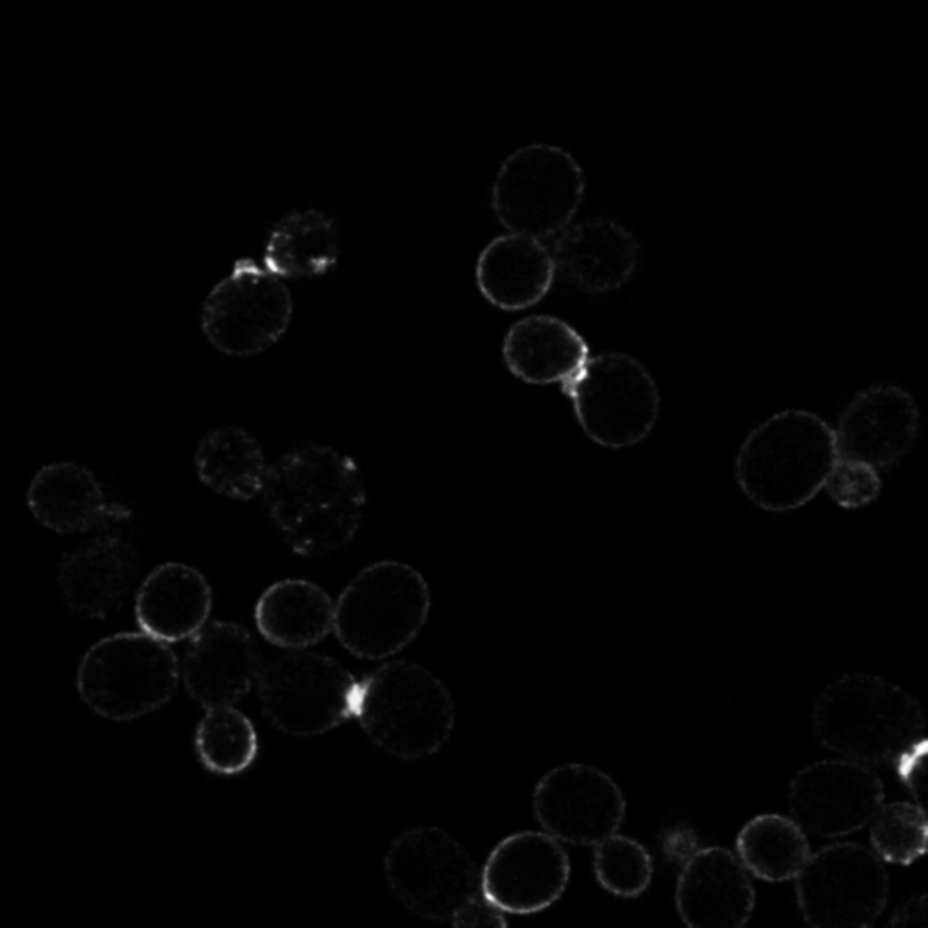

Supplement: Supplementary file 19 — EV Figures Source Data [file 44318_2025_618_MOESM19_ESM.zip › Expanded View Figures/Figure EV2/EV2F/GFP-D4H FM4-64 t0.tif]

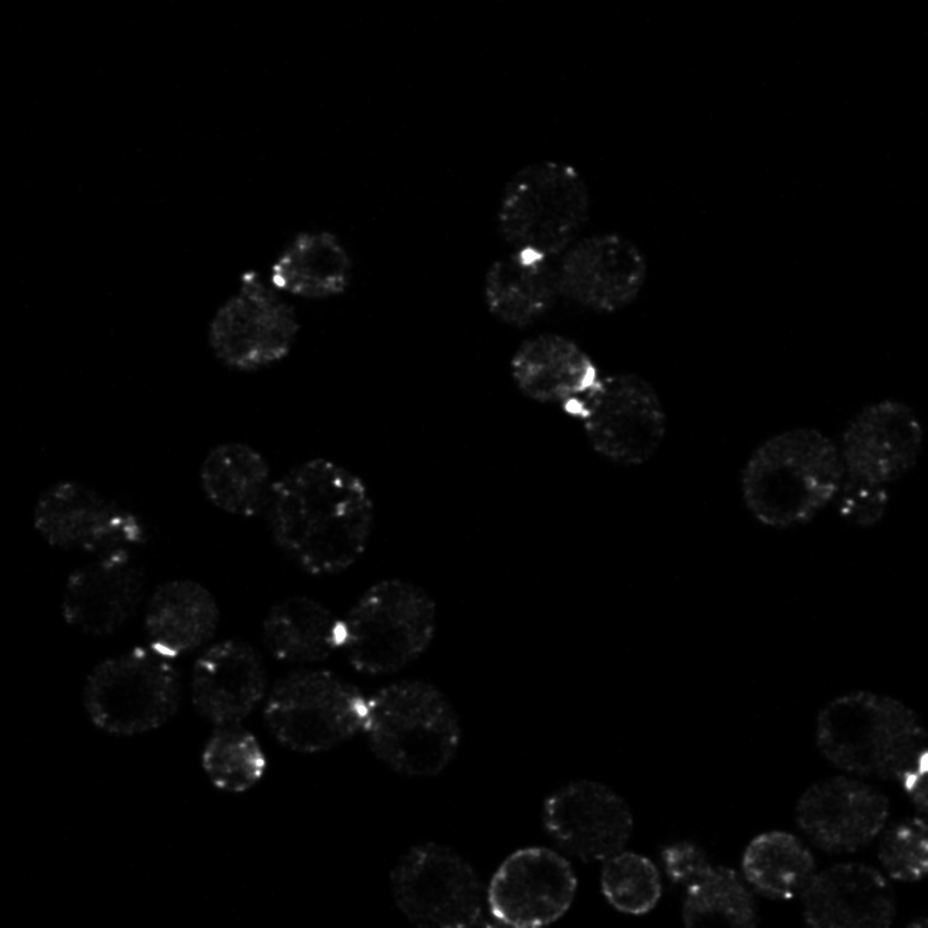

Supplement: Supplementary file 19 — EV Figures Source Data [file 44318_2025_618_MOESM19_ESM.zip › Expanded View Figures/Figure EV2/EV2F/GFP-D4H FM4-64 t2.tif]

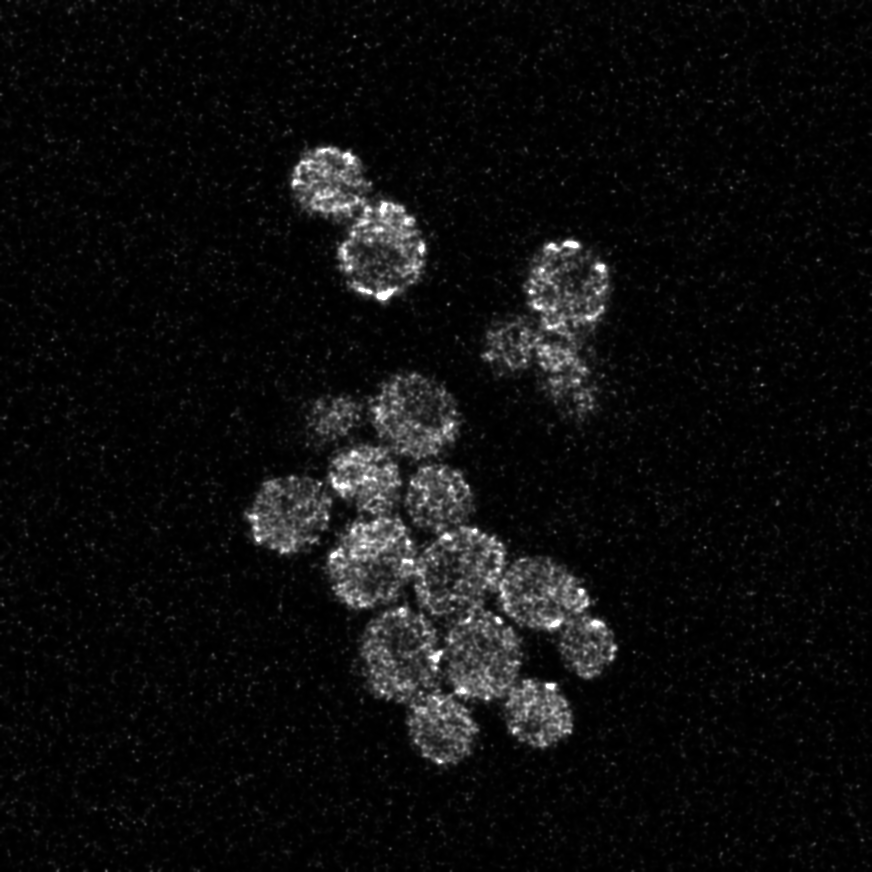

Supplement: Supplementary file 19 — EV Figures Source Data [file 44318_2025_618_MOESM19_ESM.zip › Expanded View Figures/Figure EV2/EV2K/GFP-Lam2 2xPH t0.tif]

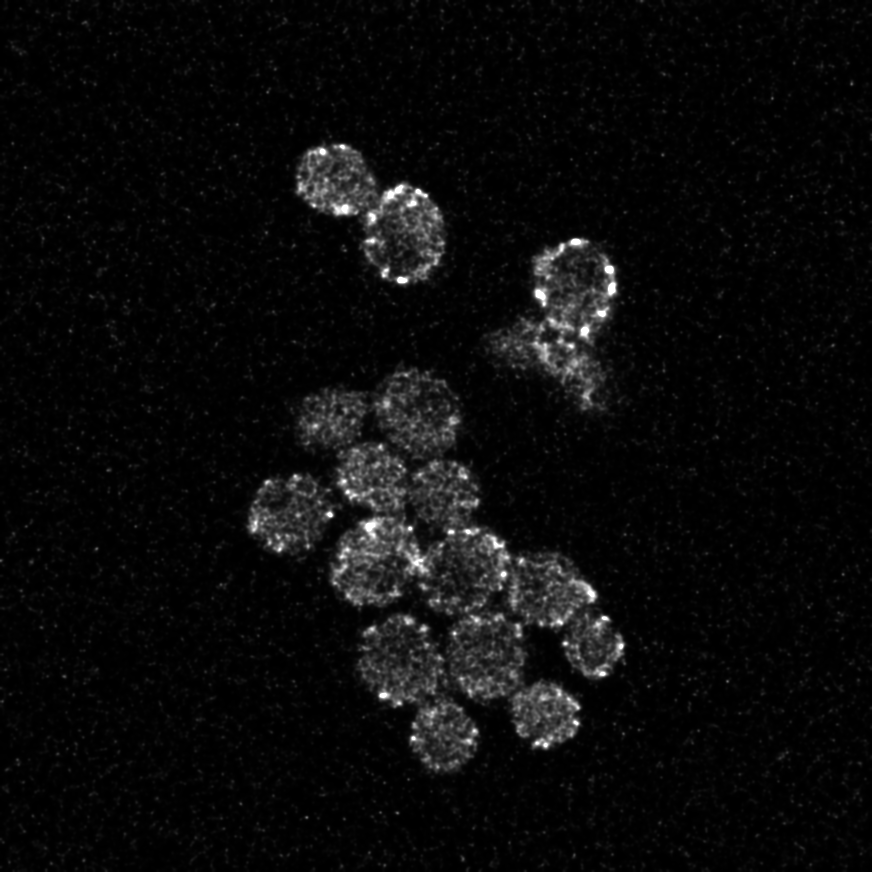

Supplement: Supplementary file 19 — EV Figures Source Data [file 44318_2025_618_MOESM19_ESM.zip › Expanded View Figures/Figure EV2/EV2K/GFP-Lam2 2xPH t5.tif]

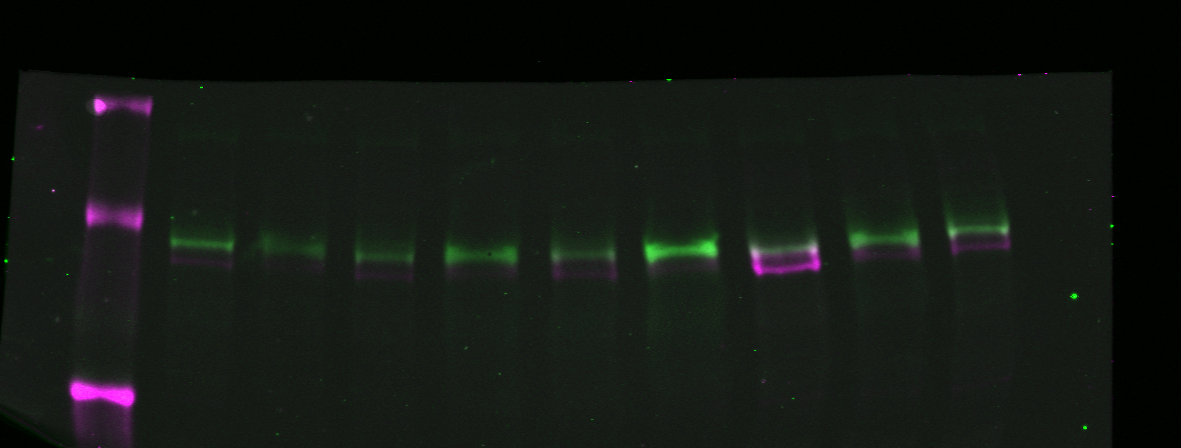

Supplement: Supplementary file 19 — EV Figures Source Data [file 44318_2025_618_MOESM19_ESM.zip › Expanded View Figures/Figure EV2/EV2M/uncropped WB images/Statin WB.tif]

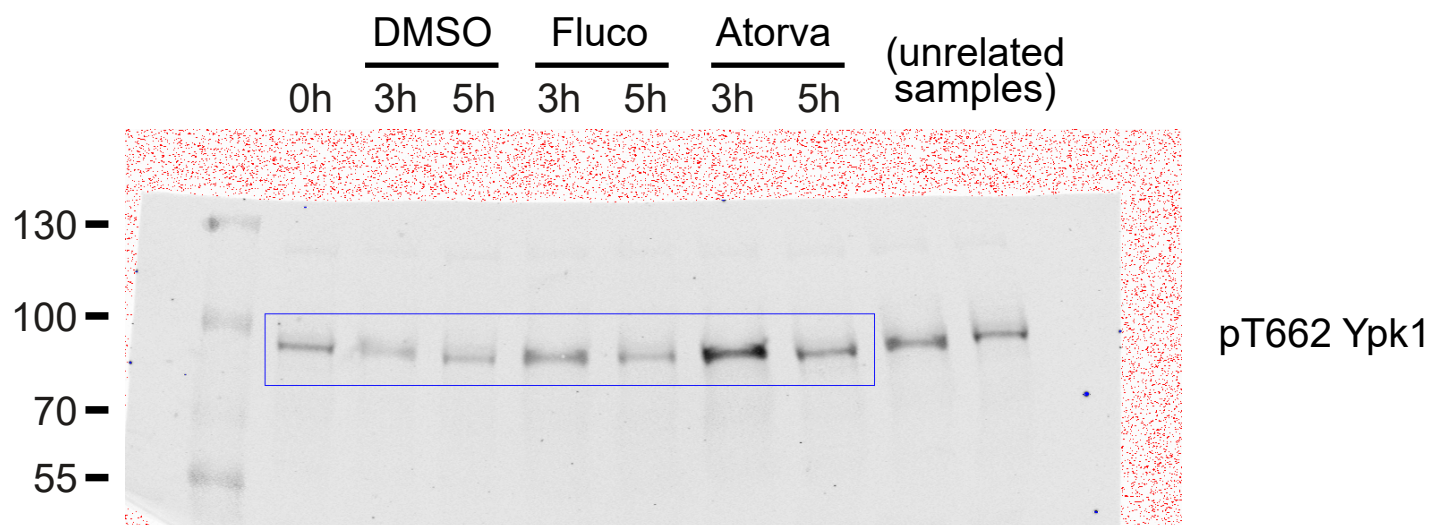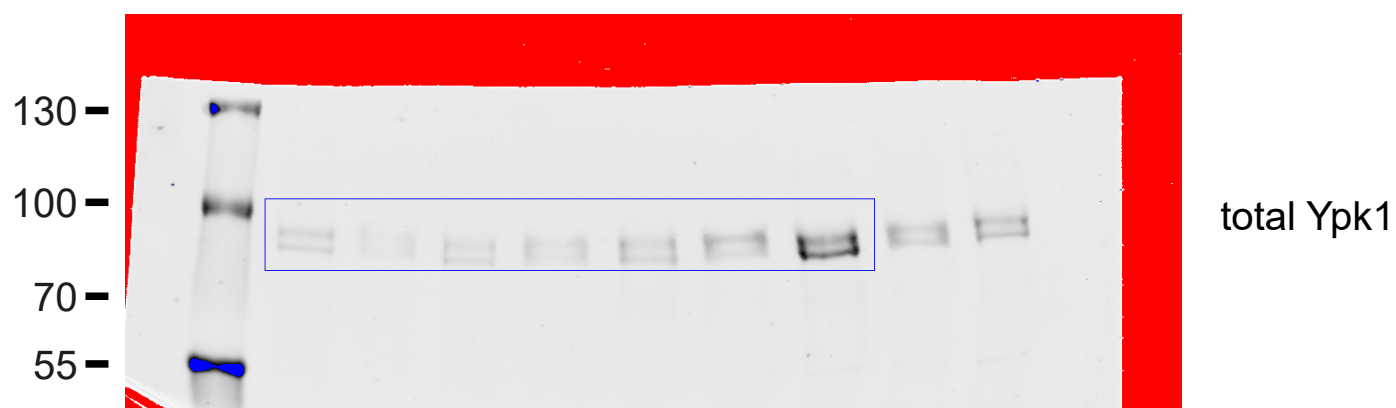

Supplement: Supplementary file 19 — EV Figures Source Data [file 44318_2025_618_MOESM19_ESM.zip › Expanded View Figures/Figure EV2/EV2M/uncropped WBs annotated.pdf]

# Experiment 20240502

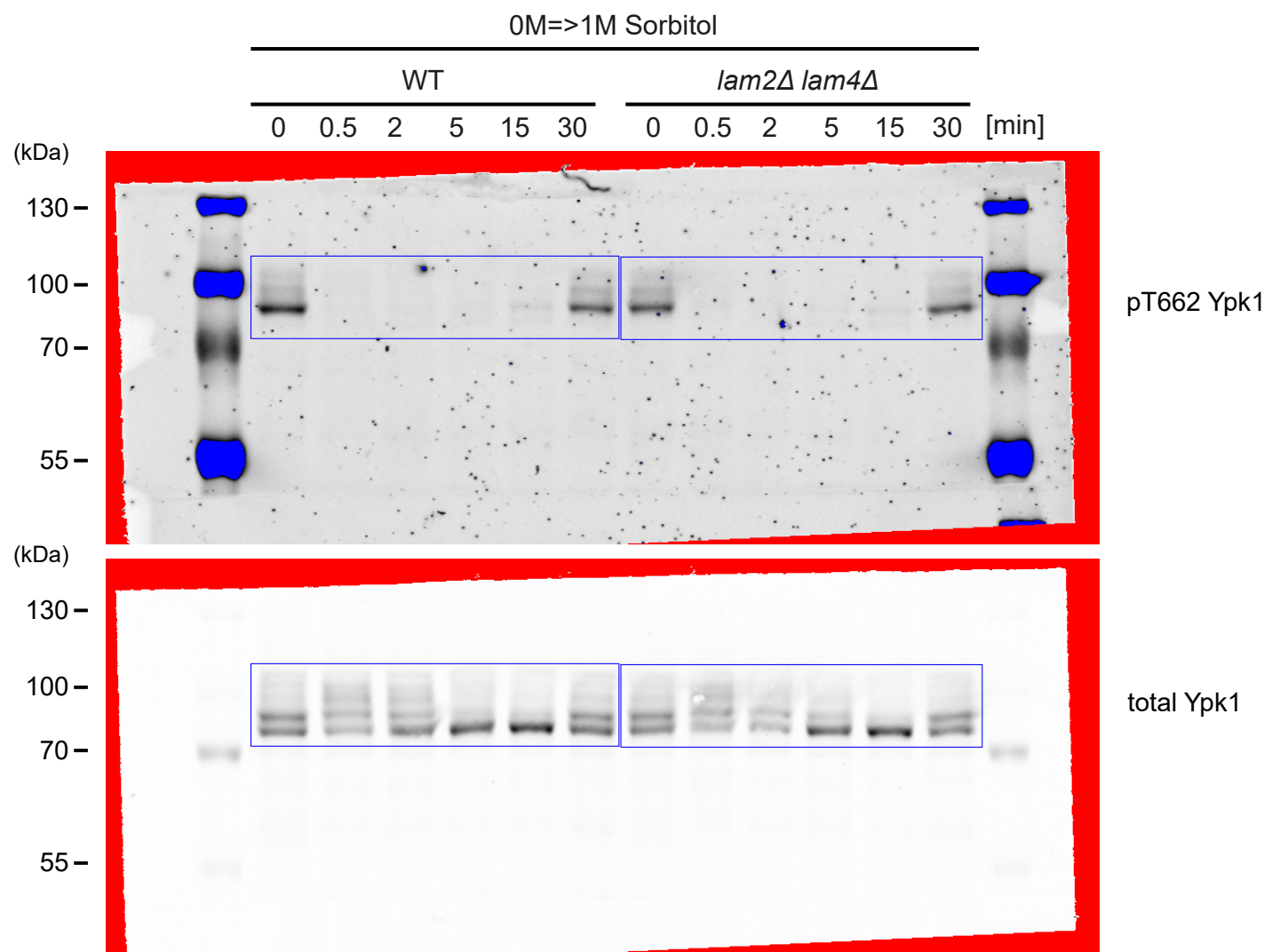

Supplement: Supplementary file 19 — EV Figures Source Data [file 44318_2025_618_MOESM19_ESM.zip › Expanded View Figures/Figure EV4/EV4A/EV4A uncropped WB labelled.pdf]

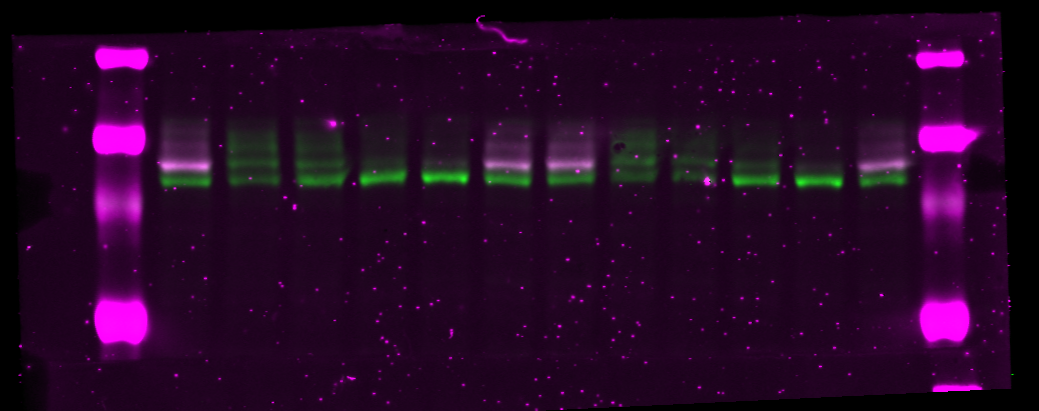

Supplement: Supplementary file 19 — EV Figures Source Data [file 44318_2025_618_MOESM19_ESM.zip › Expanded View Figures/Figure EV4/EV4A/uncropped WB images/20240502.tif]

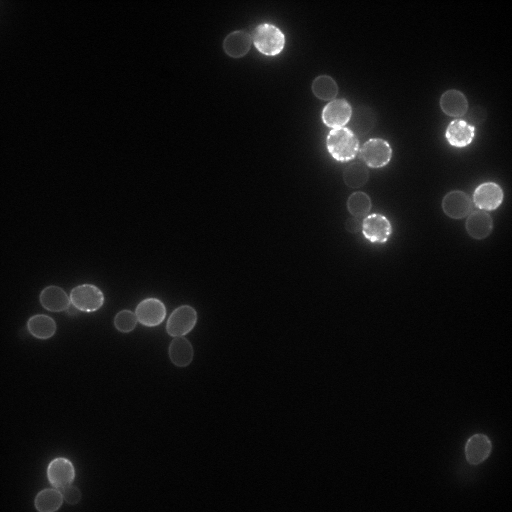

Supplement: Supplementary file 19 — EV Figures Source Data [file 44318_2025_618_MOESM19_ESM.zip › Expanded View Figures/Figure EV4/EV4C/lam baseline1_t0.tif]

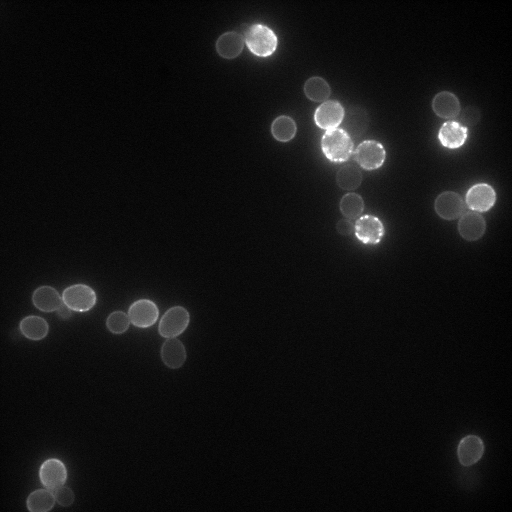

Supplement: Supplementary file 19 — EV Figures Source Data [file 44318_2025_618_MOESM19_ESM.zip › Expanded View Figures/Figure EV4/EV4C/lam baseline1_t10.tif]

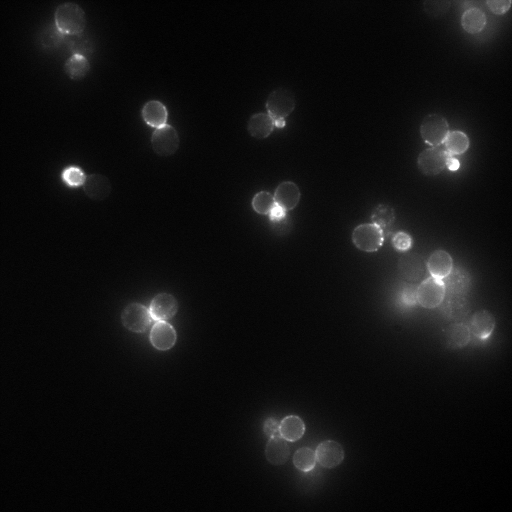

Supplement: Supplementary file 19 — EV Figures Source Data [file 44318_2025_618_MOESM19_ESM.zip › Expanded View Figures/Figure EV4/EV4C/WT baseline1_t0.tif]

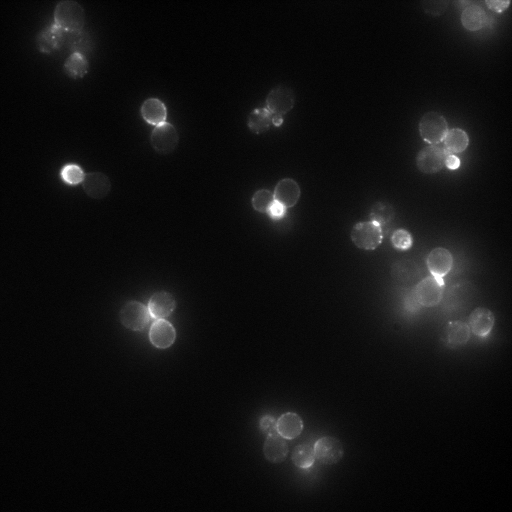

Supplement: Supplementary file 19 — EV Figures Source Data [file 44318_2025_618_MOESM19_ESM.zip › Expanded View Figures/Figure EV4/EV4C/WT baseline1_t10.tif]
