# Supplementary material for: A dynamic feedback loop between retrograde sterol transport and TORC2 controls adaptation of the plasma membrane to stress
Source: EMBO J. 2025 Nov 13;44(24):7541–64. doi: 10.1038/s44318-025-00618-7 (PMC12705765; doi:10.1038/s44318-025-00618-7)
Supplement: Supplementary file 20 — Expanded View Figures [file 44318_2025_618_MOESM20_ESM.pdf]

Expanded View Figures

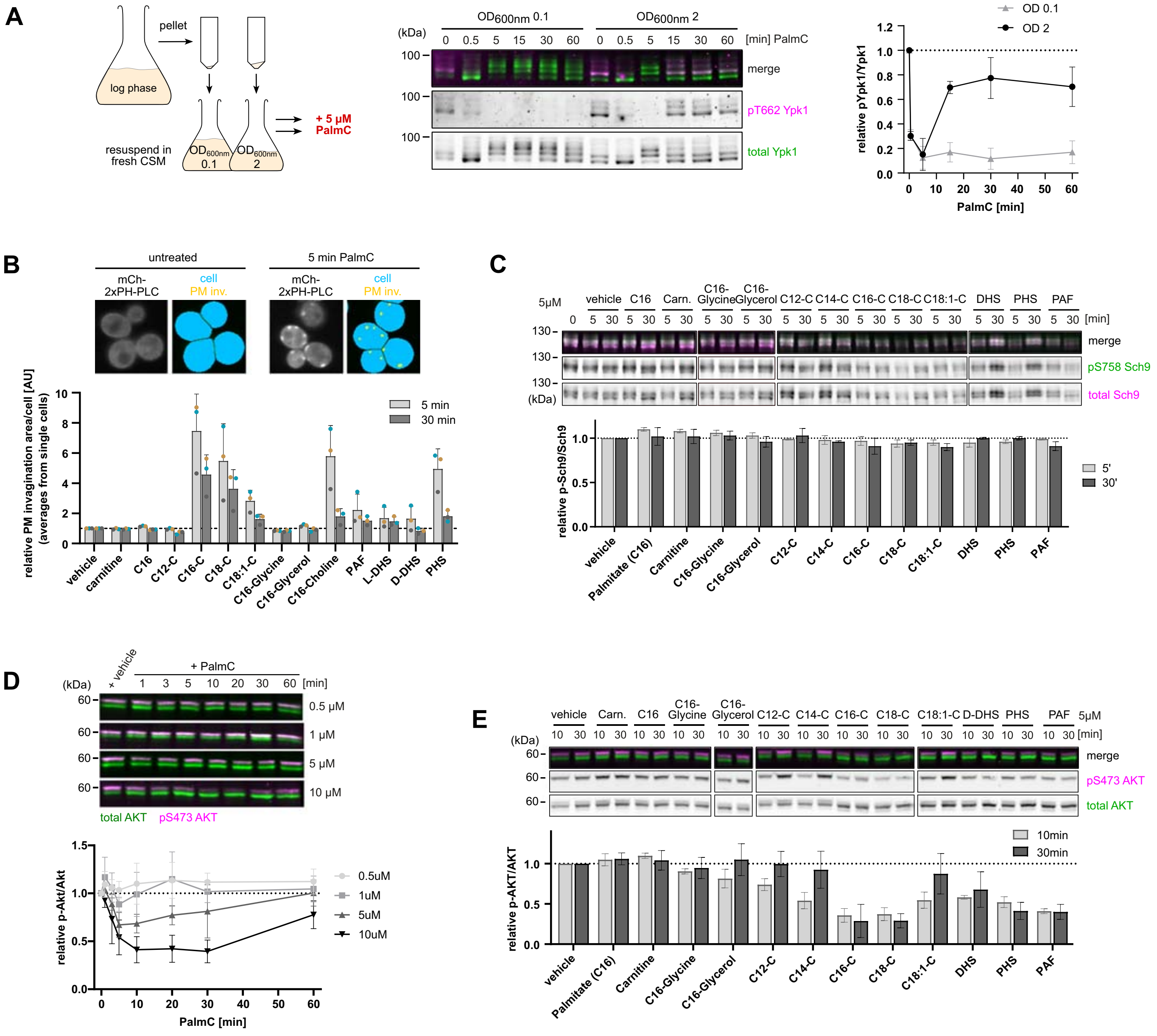

Figure EV1. Additional characterization of the effects of small amphipaths in yeast and mammalian cells.

(**A**) Western blot analysis of the effect of different yeast culture densities on PalmC-induced inhibition of TORC2. WT cells were pelleted and resuspended in fresh media to either OD 0.1 or OD 2 before treatment with 5 µM PalmC, and TORC2 activity was assessed by relative phosphorylation of Ypk1. Mean and SD of $N = 3$ independent experiments. (**B**) PM invagination screen in fixed WT cells expressing mCh-2xPH$^{PLC\delta}$. Cells were treated with the indicated substances at 5 µM, and samples were taken at the indicated timepoints and fixed. Representative images and segmentations of untreated cells and cells treated with PalmC for 5 min are shown. The plot shows the relative cell area fraction occupied by PM invaginations under different treatments. Determined values for each condition were normalized to the same vehicle timepoint for each experiment. Mean and SD of $N = 3$ independent experiments. (**C**) Western blot analysis showing the effect of different PalmC derivatives on TORC1 activity. WT cells were treated with indicated substances at 5 µM for 5 or 30 min, and TORC1 activity was assessed by relative phosphorylation of Sch9. Mean and SD of $N = 3$ independent experiments. (**D**, **E**) Western blot analysis of mTORC2 activity in HBEC3-KT cells. Cells were treated with (**D**) different concentrations of PalmC, or (**E**) with the indicated PalmC derivatives at 5 µM, and mTORC2 activity was assessed by relative phosphorylation of AKT. Mean and SD of $N = 3$ independent experiments. Source data are available online for this figure.

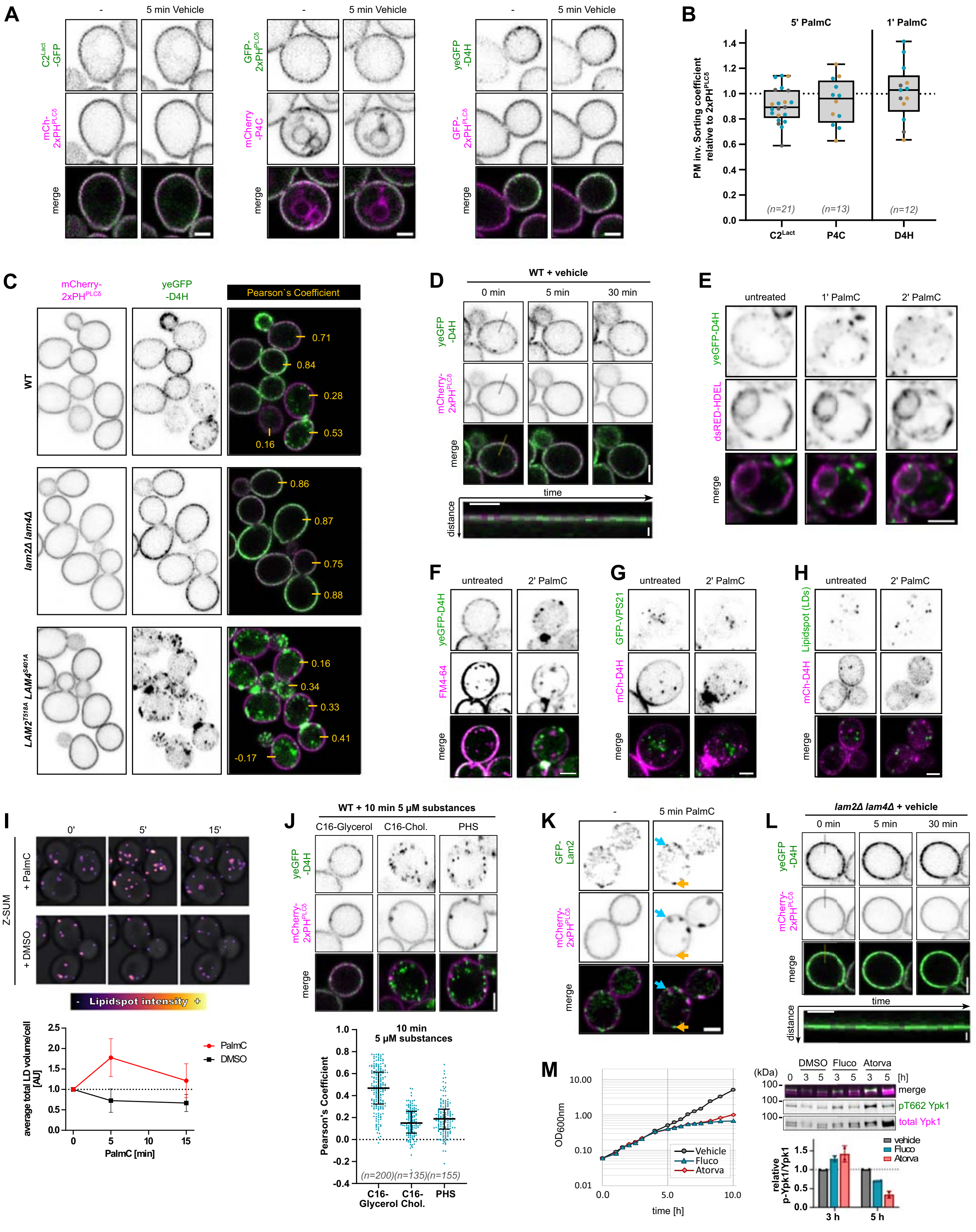
A
5 min Vehicle
C2Lact-GFP
mCh-2xPHPLCδ
GFP-2xPHPLCδ
mCherry-P4C
yeGFP-D4H
merge
B
5' PalmC
1' PalmC
PM inv. Sorting coefficient relative to 2xPHPLCδ
(n=21) (n=13) (n=12)
C2Lact P4C D4H
C
mCherry-2xPHPLCδ
yeGFP-D4H
Pearson`s Coefficient
WT
lam2Δ lam4Δ
LAM2T518A LAM4S401A
D
WT + vehicle
0 min 5 min 30 min
time
distance
E
untreated 1' PalmC 2' PalmC
dsRED-HDEL
F
FM4-64
G
GFP-VPS21
mCh-D4H
H
Lipidspot (LDs)
I
Z-SUM
+ PalmC
+ DMSO
Lipidspot intensity
average total LD volume/cell [AU]
PalmC [min]
J
WT + 10 min 5 µM substances
C16-Glycerol C16-Chol. PHS
10 min 5 µM substances
Pearson's Coefficient
(n=200)(n=135)(n=155)
K
5 min PalmC
GFP-Lam2
L
lam2Δ lam4Δ + vehicle
M
OD600nm
time [h]
Vehicle
Fluco
Atorva
DMSO Fluco Atorva
(kDa)
pT662 Ypk1
total Ypk1
relative p-Ypk1/Ypk1
3 h 5 h

Figure EV2. Expanded observations with FLAREs on the effects of PalmC and sterol depletion in yeast cells.

(**A**) Live-cell fluorescence microscopy of yeast cells expressing a PI(4,5)P2 reporter (GFP- or mCh-2xPH$^{PLC\delta}$) along with a phosphatidylserine (PS) reporter (C2^{Lact}-GFP), a PI4P reporter (mCh-P4C), or a free ergosterol reporter (yeGFP-D4H). Cells were treated with vehicle for 5 min. Scale bar = 2 µm. (**B**) Relative enrichment (sorting coefficients) of C2^{Lact}-GFP and mCh-P4C in PM invaginations after 5 min 5 µM PalmC treatment (left panel), or of yeGFP-D4H after 1 min 5 µM PalmC treatment (right panel), calculated using 2xPH$^{PLC\delta}$ as a reference. Single values from independent experiments (color coded) are plotted together with median, 25–75% interquartile range (box), and min-max range (whiskers). (**C**) Representative images showing yeGFP-D4H distribution and corresponding Pearson's Correlation Coefficients as measure of colocalisation with mCh-2xPH$^{PLC\delta}$ across the population in WT (top), *lam2Δ lam4Δ* (middle) or *LAM2*^{T518A} *LAM4*^{S401A} (bottom) cells. (**D**) Live cell fluorescence microscopy of free ergosterol (yeGFP-D4H) and PI(4,5)P2 (mCh-2xPH$^{PLC\delta}$) in WT yeast cells before, and at indicated timepoints after the addition vehicle. Scale bar = 2 µm. The kymograph (bottom panel) depict yeGFP-D4H distribution relative to mCh-2xPH$^{PLC\delta}$-marked PM along the specified line over 30 min, at 1-min intervals. Scale bars: $x = 5$ min, $y = 0.5$ µm. (**E–H**) Live-cell fluorescence microscopy of yeast cells expressing a free ergosterol reporter (yeGFP- or mCherry-D4H), imaged before and 1–2 min after treatment with 5 µM PalmC. Cells are additionally also either (**E**) expressing dsRED-HDEL to mark ER (MAX projection of 3 equatorial slices, corresponding to a section of ~1.3 µm thickness), (**F**) stained with FM4-64 and incubated for 10 min to mark early endosomes (single equatorial slice) (**G**) overexpressing GFP-Vps21 to mark early endosomes (single equatorial slice), or (**H**) stained with Lipidspot™ 488 to mark lipid droplets (single equatorial slice). Scale bar = 2 µm. (**I**) Live-cell fluorescence microscopy of yeast cells stained with Lipidspot™ 488 to mark lipid droplets, and imaged before and after addition of 5 µM PalmC or vehicle at the indicated timepoints. Z-SUM projections of Lightning-deconvolved z stacks (merge brightfield and Lipidspot) are shown. Total lipid droplet volumes were determined in single cells, averaged, and the relative change to t0 was calculated. Mean and SD from $N = 3$ independent experiments. (**J**) Live cell fluorescence microscopy of free ergosterol (yeGFP-D4H) and PI(4,5)P2 (mCh-2xPH$^{PLC\delta}$) in yeast cells treated with indicated PalmC derivatives at 5 µM for 10 min. Scale bar = 2 µm. Scatter plots show the colocalization between the two probes, data points represent individual cells, plotted with median and 25%-75% interquartile range. Values from one representative experiment are shown. (**K**) Live cell fluorescence microscopy of yeast cells expressing a PI(4,5)P2 reporter (mCh-2xPH$^{PLC\delta}$) along with GFP-Lam2, before and after 5 µM PalmC treatment for 5 min. Scale bar = 2 µm. (**L**) Live cell fluorescence microscopy of free ergosterol (yeGFP-D4H) and PI(4,5)P2 (mCh-2xPH$^{PLC\delta}$) in *lam2Δ lam4Δ* yeast cells before, and at indicated timepoints after the addition vehicle. Scale bar = 2 µm. The kymograph (bottom panel) depict yeGFP-D4H distribution relative to mCh-2xPH$^{PLC\delta}$-marked PM along the specified line over 30 min, at 1-min intervals. Scale bars: $x = 5$ min, $y = 0.5$ µm. (**M**) Growth curve (left), and phospho-Ypk1 Western blot as readout for TORC2 activity (right), as measured simultaneously in logarithmically growing WT yeast cells treated either with 100 µM Atorvastatin, or 100 µM Fluconazole, or vehicle. The growth curve is a representative example from $N = 1$ experiment. The bar graph depicts relative Ypk1 phosphorylation; the values from $N = 2$ independent experiments are plotted with mean and SD. Source data are available online for this figure.

© The Author(s)

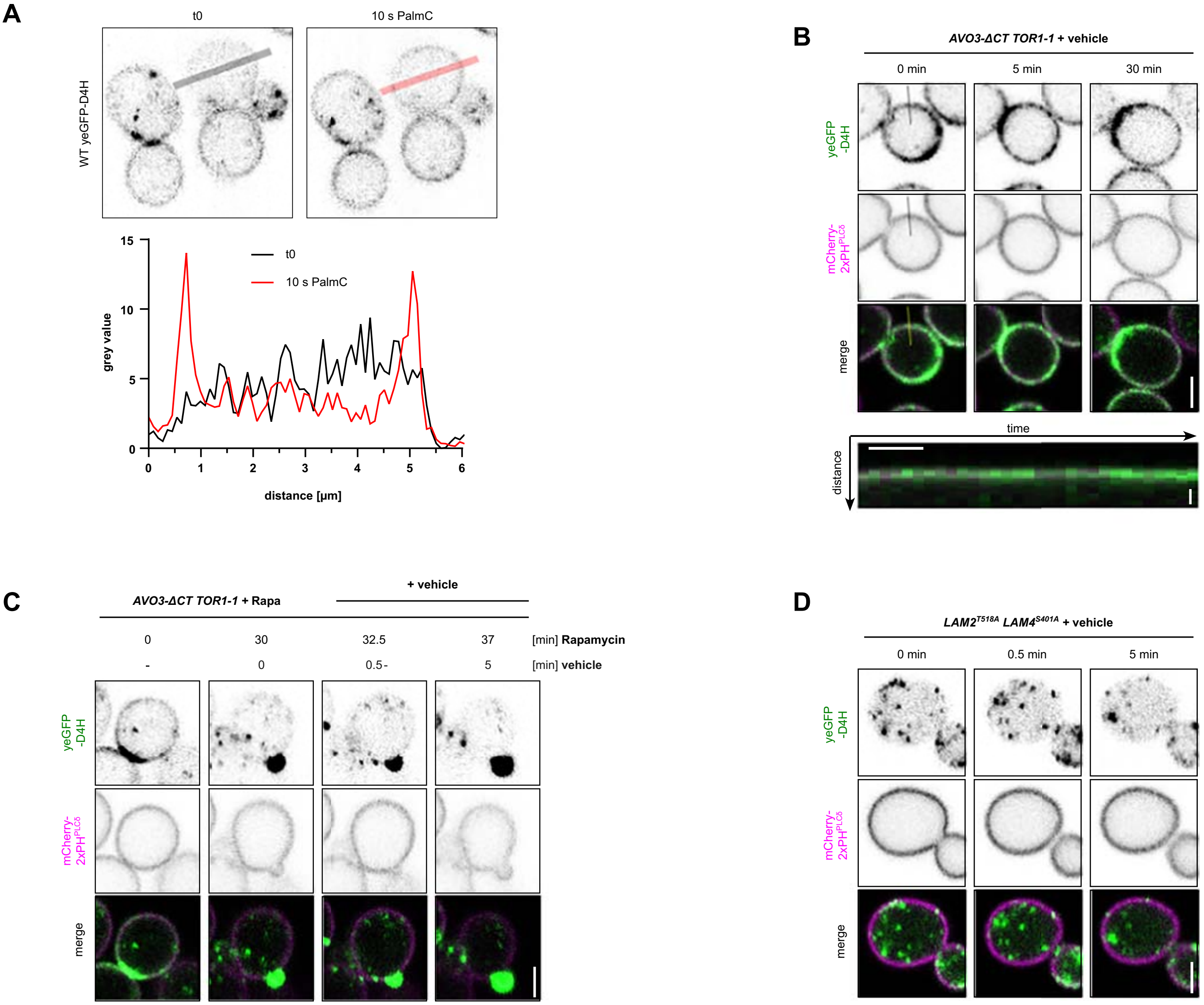

Figure EV3. PalmC increases free PM ergosterol in WT cells, and vehicle control images for Fig. 3.

(**A**) Live cell fluorescence microscopy of free ergosterol (yeGFP-D4H) in WT cells before and 10 s after 5 µM PalmC addition to WT cells. The line plot represents the average signal intensity of yeGFP-D4H along the specified line. (**B–D**) Live cell fluorescence microscopy of free ergosterol (yeGFP-D4H) and PI(4,5)P2 (mCh-2xPH$^{PLC\delta}$) (**B**) in *AVO3-ΔCT TOR1-1* cells before and after addition of 200 nm Rapamycin or (**C**) before and after 30 min pretreatment with 200 nM Rapamycin, followed by addition of vehicle, or (**D**) in *LAM2^{T518A} LAM4^{S401A}* cells before and after addition of vehicle. Scale bar = 2 µm. The kymograph (bottom panel, if present) depicts yeGFP-D4H distribution relative to mCh-2xPH$^{PLC\delta}$-marked PM along the specified line over 30 min, at 1-min intervals. Scale bars: $x = 5$ min, $y = 0.5$ µm. Source data are available online for this figure.

 © The Author(s)

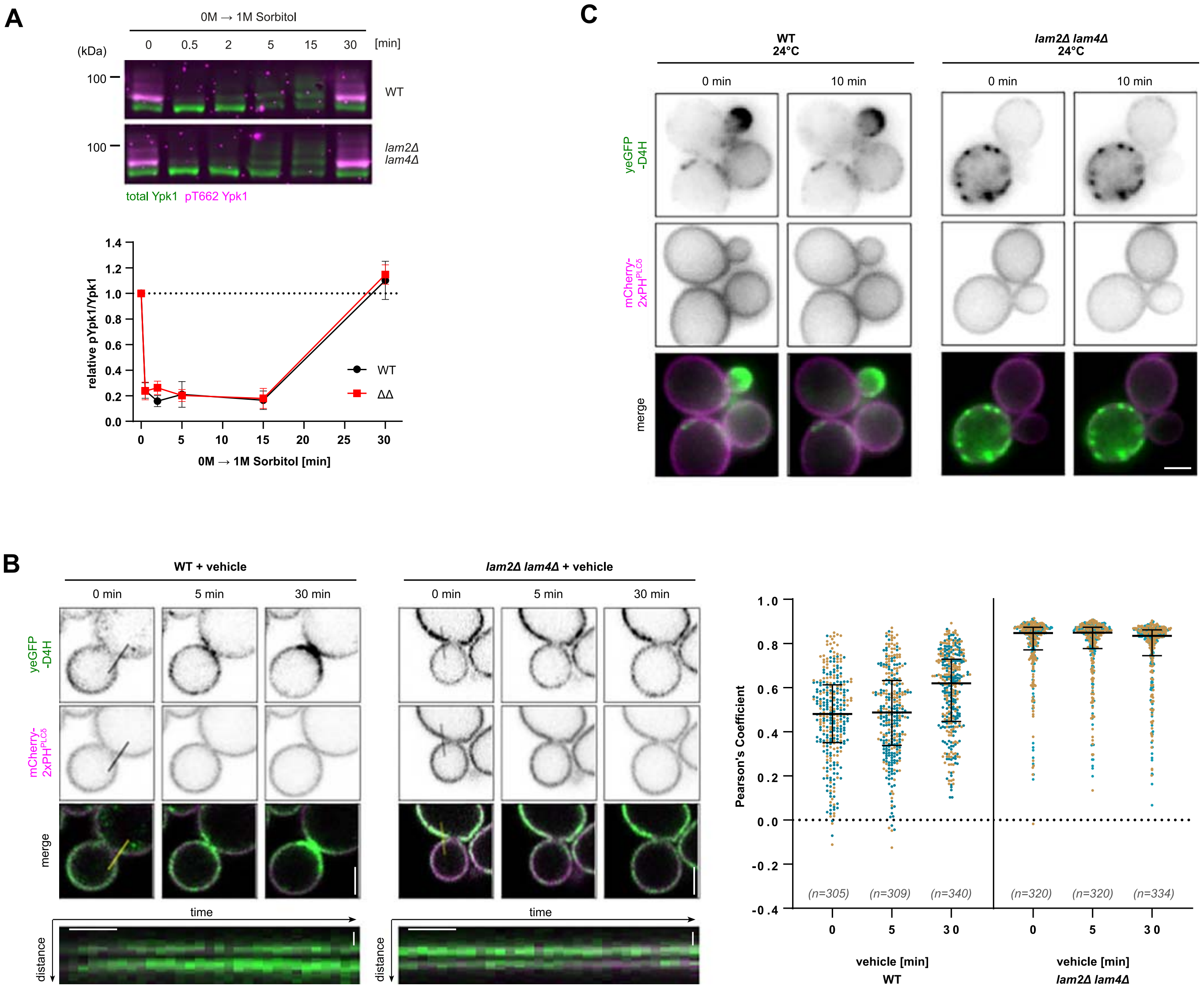

Figure EV4. TORC2 activity during severe hyperosmotic shock, and mock/vehicle controls for Fig. 4.

(**A**) Western blot analysis of TORC2 activity in yeast cells. WT and *lam2Δ lam4Δ* cells were treated with 1 M Sorbitol and TORC2 activity was assessed by relative phosphorylation of Ypk1. Mean and SD of N = 3 independent experiments. (**B**, **C**) Live cell fluorescence microscopy of free ergosterol (yeGFP-D4H) and PI(4,5)P2 (mCh-2xPH$^{PLC\delta}$) in (**B**) WT and *lam2Δ lam4Δ* cells before and after addition of vehicle (DMSO). Scale bar = 2 µm. The kymograph (bottom panel) depict yeGFP-D4H distribution relative to mCh-2xPH$^{PLC\delta}$-marked PM along the specified line over 30 min, at 1-min intervals. Scale bars: $x = 5$ min, $y = 0.5$ µm. Scatter plots (if present) show the colocalization between the two probes before and at the indicated timepoints post-treatment. Data points represent individual cells, plotted with median and interquartile range. Different colors represent data from independent experiments. (**C**) WT and *lam2Δ lam4Δ* cells before and after 10 min of imaging at the idle growth temperature of 24 °C. Source data are available online for this figure.

© The Author(s)

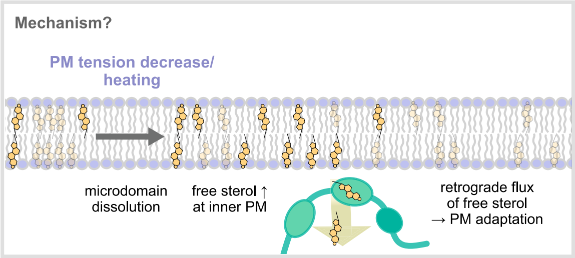

Figure EV5. Speculative mechanism for PM stress recovery by sterol transport.

Hyperosmotic shock and branched actin/endocytosis inhibition by CK-666 could both cause a dissolution of PM microdomains via a loss of PM tension. This would increase free PM sterols, which in combination with TORC2 inhibition could lead to an increased sterol retrograde transport through Lam2/4. Heat shock on the other hand could increase free PM sterols by thermic microdomain disruption.

 © The Author(s)
