# Supplementary material for: A dynamic feedback loop between retrograde sterol transport and TORC2 controls adaptation of the plasma membrane to stress
Source: EMBO J. 2025 Nov 13;44(24):7541–64. doi: 10.1038/s44318-025-00618-7 (PMC12705765; doi:10.1038/s44318-025-00618-7)

# Experiment 20250522

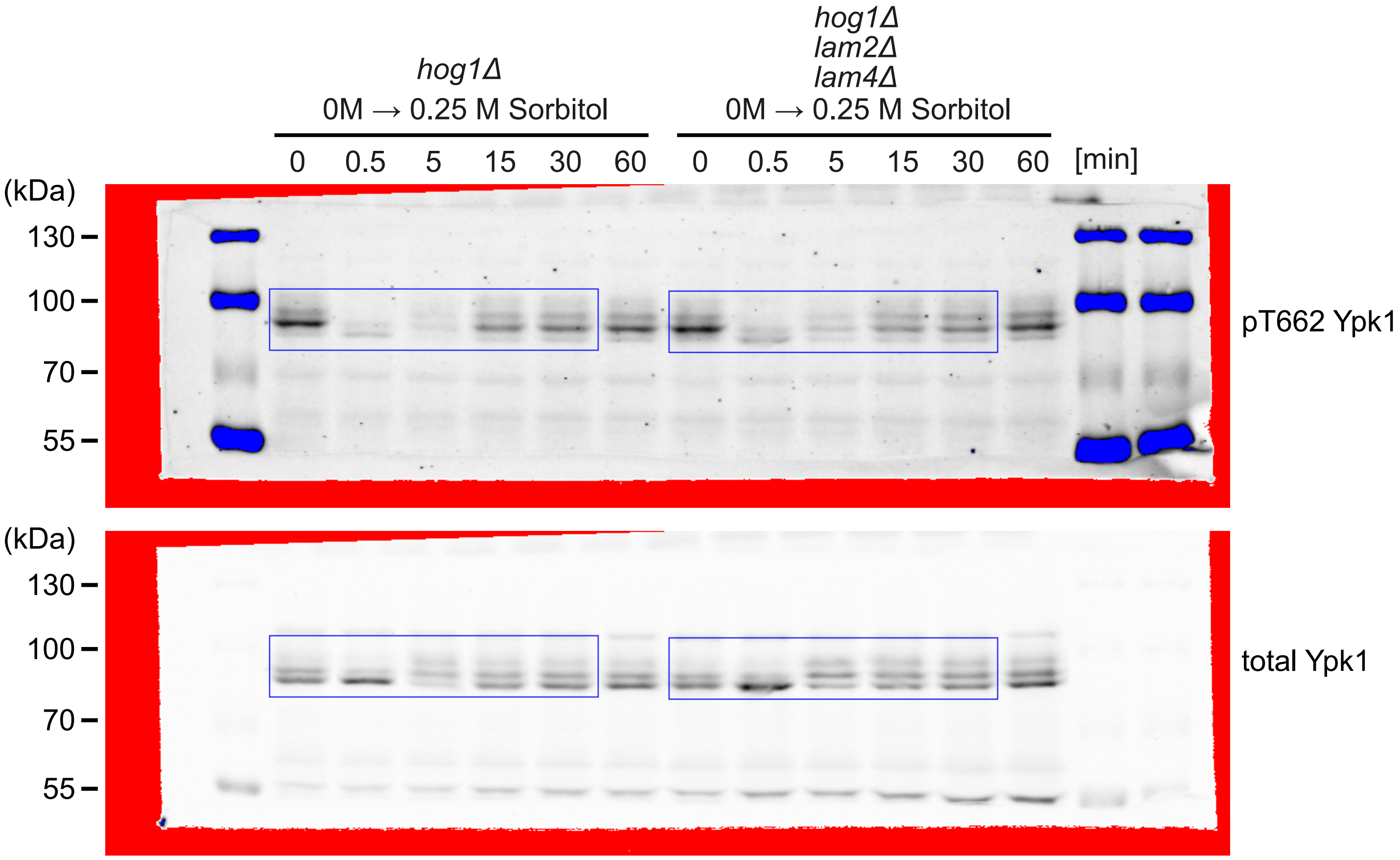

Supplement: Supplementary file 18 — Source data Fig. 4 [file 44318_2025_618_MOESM18_ESM.zip › Figure 4/4B/Uncropped WBs annotated.pdf]
